# Thermodynamic coupling of the tandem RRM domains of hnRNP A1 underlie its pleiotropic RNA binding functions

**DOI:** 10.1126/sciadv.adk6580

**Published:** 2024-07-10

**Authors:** Jeffrey D. Levengood, Davit Potoyan, Srinivasa Penumutchu, Abhishek Kumar, Qianzi Zhou, Yiqing Wang, Alexandar L. Hansen, Sebla Kutluay, Julien Roche, Blanton S. Tolbert

**Affiliations:** ^1^ Department of Biochemistry and Biophysics, University of Pennsylvania, Philadelphia, PA 19104, USA.; ^2^ Department of Chemistry, Iowa State University, Ames, IA 50011, USA.; ^3^ Department of Molecular Microbiology, Washington University School of Medicine, St. Louis, MO 63110, USA.; ^4^ New York University Grossman School of Medicine, New York, NY 10016, USA.; ^5^ CCIC and Gateway NMR Facility, The Ohio State University, Columbus, OH 43210, USA.; ^6^ Roy J. Carver Department of Biochemistry, Biophysics and Molecular Biology, Iowa State University, Ames, IA 50011, USA.; ^7^ Howard Hughes Medical Institute, Chevy Chase, MD 20815, USA.

## Abstract

The functional properties of RNA binding proteins (RBPs) require allosteric regulation through interdomain communication. Despite the importance of allostery to biological regulation, only a few studies have been conducted to describe the biophysical nature by which interdomain communication manifests in RBPs. Here, we show for hnRNP A1 that interdomain communication is vital for the unique stability of its amino-terminal domain, which consists of two RNA recognition motifs (RRMs). These RRMs exhibit drastically different stability under pressure. RRM2 unfolds as an individual domain but remains stable when appended to RRM1. Variants that disrupt interdomain communication between the tandem RRMs show a significant decrease in stability. Carrying these mutations over to the full-length protein for in vivo experiments revealed that the mutations affected the ability of the disordered carboxyl-terminal domain to engage in protein-protein interactions and influenced the protein’s RNA binding capacity. Collectively, this work reveals that thermodynamic coupling between the tandem RRMs of hnRNP A1 accounts for its allosteric regulatory functions.

## Teaser

hnRNP A1 is an allosterically regulated RNA binding proteins.

## INTRODUCTION

**Fig. 1.**
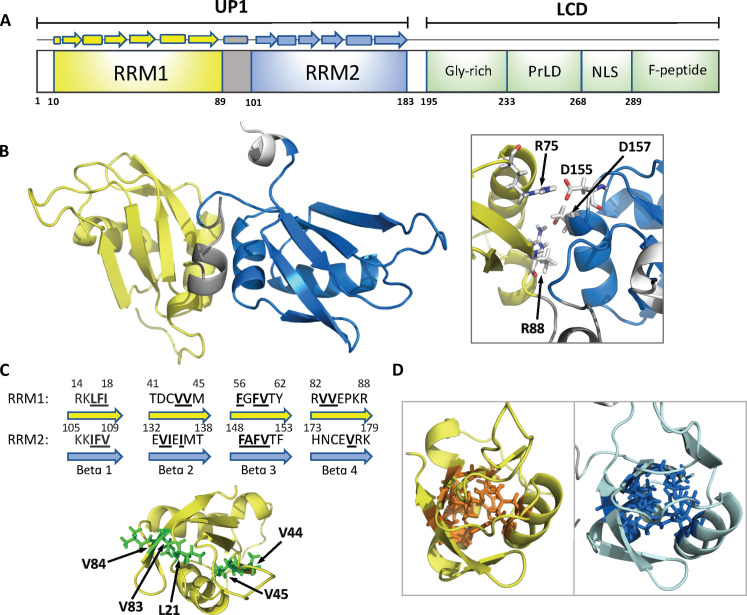
Overview of hnRNP A1 domains and UP1 structure. (**A**) Schematic representation of hnRNP A1-A sequence with the N-terminal UP1 domain encompassing two RRMs (RRM1 and RRM2) and the C-terminal LCD encompassing the glycine-rich region (Gly-rich), prion-like domain (prLD), nuclear localization signal (NLS), and phosphopeptide (F-peptide). (**B**) 3D structure of the UP1 domain (PDB 1U1R) showing RRM1 in yellow, RRM2 in blue, and the linker between the two RRMs in gray. The insert highlights the two salt bridges (R75:D155 and R88:D157) at the interface between RRM1 and RRM2. (**C**) Top: Sequence comparison of the four-stranded β sheet found in RRM1 and RRM2. Nonpolar amino acids composing the core of each RRM are shown in bold fonts. Bottom: Close-up of the hydrophobic patch present in the core of RRM1. (**D**) Close-up view of the RRMs from UP1 highlighting the higher packing density of RRM1 core (left) compared to RRM2 (right).

The heterogeneous nuclear ribonucleoprotein A1 (hnRNP A1) is a ubiquitous RNA binding protein that regulates RNA metabolism both under normal and pathological cellular conditions, including viral infections (*1*–*3*). As a general regulator of RNA biology, hnRNP A1 engages with transcripts from the moment they are synthesized, processed to maturity, exported from the nucleus, and translated into protein products (*1*, *4*). HnRNP A1 imparts its broad functions via a domain organization that consists of tandem RNA recognition motifs (RRMs), collectively known as UP1 (unwinding protein 1), at its N terminus and a C-terminal low complexity domain (LCD_A1_) that is mostly disordered but engages in heterotypic protein-protein and protein-RNA interactions (Fig. 1A) (*4*–*6*). Efforts to reconcile the pleiotropic functions of hnRNP A1 have led to multiple structures of its UP1 domain, each showing different mechanisms of RNA or DNA recognition (*7*–*10*). These structures along with the broad RNA binding landscape of hnRNP A1 suggests that the protein binds its various targets via idiosyncratic mechanisms (*1*). The underlying physicochemical properties by which hnRNP A1 achieves cognate RNA recognition remain incompletely understood, however. Adding to the hnRNP A1-RNA recognition paradox, its RRM domains are highly similar both in sequence composition and three-dimensional (3D) structure, yet they exhibit contextual differences in RNA binding properties (*9*, *11*, *12*).

A previous crystal structure from our lab demonstrated that hnRNP A1 can bind to RNA exclusively through its RRM1 domain and an inter-RRM linker to form complexes with 1:1 stoichiometries (*7*). Although RRM2 did not contact RNA, we demonstrated that its physical coupling to RRM1, stabilized by two salt bridge interactions (R75:D155 and R88:D157) (Fig. 1B), was necessary to confer high-affinity RNA recognition to hnRNP A1. R75 and R88 are located on the α-helical side of RRM1 α_2_ at the inter-RRM interface. Mutating these two residues to Asp to disrupt the salt bridges significantly reduced RNA binding affinity and was also accompanied by larger amplitude motions in the mutant protein (UP1^dm^) indicating destabilization (*7*). These results suggested that hnRNP A1 uses allosteric mechanisms to engage cognate RNA binding partners; however, the physicochemical basis of hnRNP A1 allostery was not obvious.

To explore the concept of hnRNP A1 allostery, we performed a series of high-pressure experiments using nuclear magnetic resonance (NMR) and small-angle x-ray scattering (SAXS) that were accompanied by ^15^N Carr-Purcell-Meiboom-Gill (CPMG) relaxation dispersion experiments. We complemented these experiments with extended molecular dynamic simulations and cross-linking immunoprecipitation (CLIP-seq) to demonstrate that the physical coupling of the RRMs of hnRNP A1 impart uniform thermodynamic stability across the surface of the UP1 domain to allosterically control its RNA binding capacity. Analysis of the RRM1 and RRM2 sequences revealed differences in the β sheet residues that serve as the hydrophobic cores of the RRM domains. Mutational transposition of these residues between RRM1 and RRM2 provided evidence for the strength of the hydrophobic cores being the source of domain stability. Notably, we found the core of RRM1 has a higher density of hydrophobic contacts than that of RRM2. Structure-based molecular dynamic simulations suggest that the difference in packing density is substantial enough to give rise to differences in terms of thermodynamic stabilities. ^15^N-CPMG experiments validated that the packing density difference between RRM1 and RRM2 manifests with distinct conformational fluctuations, whereby RRM1 is essentially rigid in solution while RRM2 shows evidence of conformational exchange on a μs-ms timescale.

To gain atomic level insights into the differential stabilities of the RRMs, we used high-pressure solution NMR spectroscopy to probe the unfolding thermodynamics of the RRM domains, in isolation and in tandem within UP1. We found that the two RRMs display stark differences in stability under pressure despite the highly similar sequences giving rise to nearly identical structures. RRM1 remains stable over a wide range of pressure while RRM2 completely unfolds at 2.5 kbar. Studies conducted on the UP1 domain demonstrate that the interdomain communication between RRM1 and RRM2 stabilizes the RRMs under high-pressure conditions. Notably, we show that mutations designed to break interdomain communication between the RRMs within UP1 or their structural transposition destabilize RRM2 without affecting the stability of the RRM1 domain.

Last, we examined the in vivo effects of allosteric regulation on RNA binding by performing CLIP-seq experiments with wild-type (WT) and the double salt bridge (R75D/R88D) variant of hnRNP A1 (A1^dm^). The consensus sequences identified by CLIP-seq for hnRNP A1 and the A1^dm^ variant were similar; however, comparative analysis of binding site occupancies across all immunoprecipitated transcripts shows notable variations. For example, hnRNP A1 shows enriched binding to introns relative to the A1^dm^ mutant, which correlates with statistically significant changes in the RNAs bound by the two proteins. The difference in binding capacity between hnRNP A1 and the A1^dm^ mutant correlates with the latter being defective in its ability to dimerize on the immunoprecipitated transcripts. When interpreted collectively, this study demonstrates that hnRNP A1 is an allosterically regulated RNA binding protein where the physical coupling of its tandem RRMs is necessary to confer functional recognition of its cognate RNA molecules. We posit that perturbations that change the degree of interdomain communication, such as posttranslational modifications (PTMs) or naturally occurring mutations, would, in turn, influence the pool of RNAs regulated by hnRNP A1.

## RESULTS

### The sequence and structure of hnRNP A1 determines its allostery

Since the determinants of hnRNP A1 allostery are reflected in its domain composition, we compared the physicochemical properties of RRM1 and RRM2. The tandem RRMs of hnRNP A1 adopt identical folds composed of a four-stranded β sheet and two α helices (Fig. 1, A and B) (*1*, *2*). High-resolution x-ray structures indicate that the tertiary structure of RRM1 is remarkably similar to that of RRM2 [Cα root mean square deviation, ~0.95 Å; Protein Data Bank (PDB) 1U1R] (*13*). Sequence alignment (fig. S1A) of the structural core regions of RRM1 (11 to 90) and RRM2 (102 to 183) (fig. S1B) revealed a percent identity for the pair of 34% (27 of 80) and a positive identity of 61% (49 of 80). While the percent identity is right at the minimal value of 30% for the determination of homology (*14*), the much higher value for positive identity reveals the two domains are very similar in chemical composition. Close examination of the amino acids composing the four antiparallel β strands reveals interesting differences between RRM1 and RRM2. For example, β2 and β4 of RRM1 each contain two successive valines that are absent in RRM2 (V44, V45, V83, and V84). These valines, together with L21 located in the loop that connects β1 and β2, form a long hydrophobic patch that is unique to RRM1 (Fig. 1C). Valines from RRM1 are replaced in RRM2 by an isoleucine and three polar and charged residues. The connecting loop also has one less hydrophobic residue, practically eliminating the hydrophobic patch.

Next, we compared the packing density of RRM1 and RRM2 by analyzing interside chain distances between residues composing their structural cores. We defined as structural core the 10 residues with lowest accessible surface area in each RRM (fig. S1A): L16, I18, L21, F34, G58, V60, Y62, V68, A71, and P86 for RRM1 and I107, V109, L121, F125, A149, V151, F153, V159, I162, and V177 for RRM2. We found that the average distance between the nearest side chain atoms within these core residues is 2.8 Å for RRM1 and 3.1 Å for RRM2, suggesting that the core of RRM1 is slightly more densely packed than that of RRM2 (Fig. 1D). To gauge whether this slight packing difference could potentially result in a difference in thermodynamic stability, we conducted a set of molecular dynamic simulations using a structure-based potential (Go-model). We ran a series of all-atom simulations at various temperatures using a native-based Go-type potential energy function that portrays a perfect funneled energy landscape (*15*, *16*). By computing the change in specific heat capacity as a function of temperature, we found that the isolated RRM2 (residues 95 to 196) has a slightly lower folding temperature compared to the isolated RRM1 (residues 1 to 105), suggesting that RRM2 may potentially be thermodynamically less stable than RRM1 (fig. S2).

### Differential μs-ms conformational dynamics reveal the coupling of the RRM domains of hnRNP A1

**Fig. 2.**
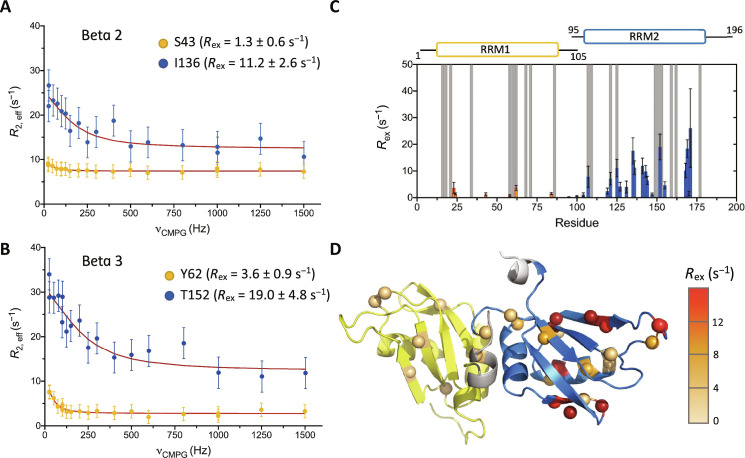
^15^N CPMG experiments conducted with the isolated RRM domains of UP1. (**A** and **B**) Representative ^15^N relaxation dispersion profiles measured for residues within (A) the second and (B) third β strand of the RRMs. Residues within RRM1 (S43 and Y62) are shown in yellow, while residues within RRM2 (I136 and T152) are shown in blue. (**C**) Chemical exchange contribution to the ^15^N transverse relaxation rate (*R*_ex_) measured for amide resonances of the isolated RRM1 (yellow) and isolated RRM2 (blue). The gray bars represent the 20 core residues with the lowest accessible surface area. (**D**) *R*_ex_ values measured on the isolated RRMs are mapped on the structure of UP1 (PDB 1U1R), showing that residues experiencing significant conformational exchange are predominantly localized within RRM2 (blue). One unassigned RRM1 peak had *R*_ex_ = 1.5 ± 0.43.

To evaluate whether the compositional differences between RRM1 and RRM2 manifest as unique physicochemical properties, we performed backbone ^15^N CPMG relaxation dispersion experiments. These experiments provide site-specific information on the contribution of microsecond to millisecond dynamic processes to the effective transverse relaxation rate constant (*R*_2_,_eff_ = *R*_2_° + *R*_ex_) (*17*, *18*). We conducted ^15^N-CPMG experiments on the UP1 domain (residues 1 to 196), isolated RRM1 (residues 1 to 105), and isolated RRM2 (residues 95 to 196) (fig. S1B). Comparison of the relaxation dispersion profiles between isolated RRM1 and RRM2 revealed notable differences for the two domains. We found conformational dynamics on the μs-ms timescale for 9 amide peaks in RRM1 and 19 amide peaks in RRM2 (Fig. 2, A and B, and fig. S3, A and B). Analysis of the *R*_ex_ data for these residues showed the magnitude of the *R*_ex_ values for the RRM2 residues were three to four times greater than those calculated for RRM1 (Fig. 2C). Since *R*_ex_ values are dependent on a variety of factors, the differences found between the two domains are less notable than the difference in the number of peaks showing significant exchange. Relative to RRM1, RRM2 has more peaks showing exchange, and at higher rates, indicating that RRM2 is more flexible. Mapping out these peaks on the protein structures revealed the areas of differential flexibility. While RRM1 peaks were dispersed throughout the domain, RRM2 peaks were clustered within β2 (I131, E135, and I136) and a noncanonical β sheet (H168-N171) between α2 and β4 (Fig. 2D). Overall, these experiments indicate that the isolated RRMs are exhibiting distinctly different conformational dynamics in solution; RRM1 being essentially rigid, while RRM2 shows evidence of conformational exchange on a μs-ms timescale.

We performed identical ^15^N CPMG experiments with UP1 to examine whether the dual domain protein showed any differences in comparison to the isolated domains. Analysis of the data revealed that only 3 residues of UP1 (fig. S3C) had significant conformational dynamics, much less than the combined 28 residues found for the two isolated domains (fig. S3, A and B). This reveals that connecting RRM1 and RRM2 via stabilization of the interdomain interface in the context of UP1 normalizes the conformational dynamics of RRM2 to the level of RRM1.

### The importance of the inter-RRM interface is revealed by pressure-induced unfolding

Next, we probed the thermodynamic stability of several variants of the UP1 domain designed to evaluate the integrity of the inter-RRM coupling. When pressurized, protein NMR spectra typically display two types of perturbations: chemical shift changes, which are due to changes in protein surface-water interface and/or small compression of protein native conformations (*19*), and crosspeak intensity changes. Loss of crosspeak intensity as a function of pressure points to major conformational transitions on a slow timescale (relative to NMR timescale) such as folding/unfolding (*20*). Here, we monitored the pressure-induced changes of crosspeak intensity of UP1 variants by recording series of 2D ^1^H-^15^N HSQC experiments from 1 bar to 2.5 kbar. In these HSQC spectra at 1 bar (fig S4, A, C, and E), the isolated RRM1 domain had 74 of the 105 amide peaks visible (the backbone chemical shifts of 60 of them were assigned), while RRM2 had 63 of the 101 amide peaks visible (52 were assigned). Fewer peaks were visible in the RRM2 spectrum due to line broadening and conformational exchange. The UP1 spectrum had 126 crosspeaks visible (114 assigned).

**Fig. 3.**
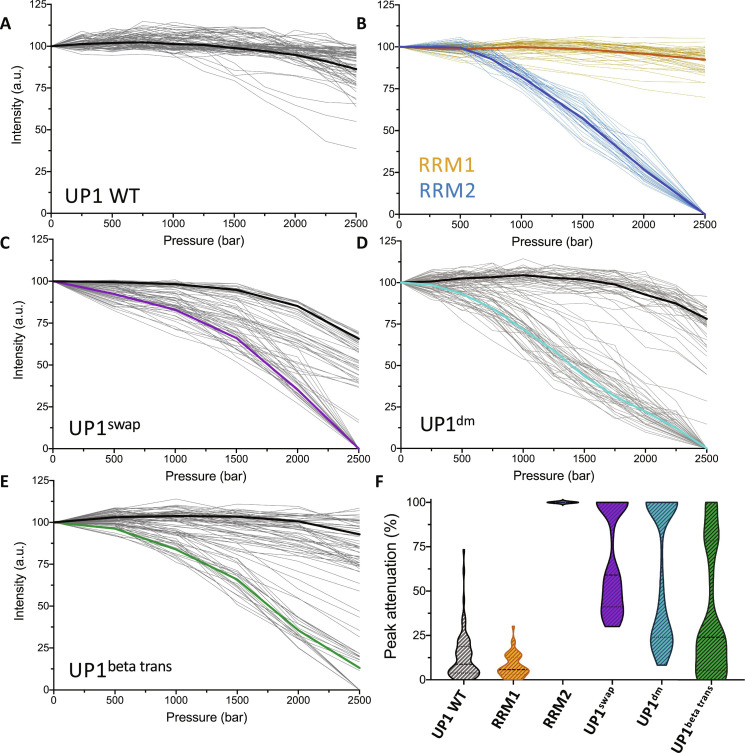
Pressure studies of UP1, its isolated RRM domains, and UP1 variants. (**A** to **E**) NMR peak intensity profiles measured as a function of pressure for individual amide resonances of (A) WT UP1, (B) isolated RRM1 (yellow) and isolated RRM2 (blue), (C) UP1^swap^, (D) UP1^dm^, and (E) UP1^beta trans^. The average peak intensity profile is shown for each variant with a bold line. (**F**) Distribution of the percentage of peak attenuation (based on the individual NMR peak intensities measured at 1 bar and at 2.5 kbar) for the amide resonances of WT UP1, isolated RRMs, and UP1 variants. a.u., arbitrary units.

Our experiments show that the UP1 domain exhibits only minor changes in crosspeak intensity as a function of pressure (~15% loss at 2.5 kbar), suggesting that UP1 remains predominantly native at high pressure (fig. S4, A and B, and Fig. 3A). Similarly, we observed no major change for the isolated RRM1 with only ~8% loss at 2.5 kbar, which indicates that RRM1 is thermodynamically stable under pressure (fig. S4, C and D, and Fig. 3B, yellow lines). By contrast, the isolated RRM2 displays a complete loss of native crosspeaks within 2.5 kbar (fig. S4, E and F, and Fig. 3B, blue lines). A decrease of native crosspeak intensity accompanies the appearance of new sets of crosspeaks with a narrow ^1^H chemical shift dispersion typically observed for protein unfolded conformations (fig. S4F). Comparison of the native and unfolded crosspeak intensity as a function of pressure indicates that the conformational transition experienced by RRM2 is characteristic of a two-state unfolding transition with a midpoint *p*_1/2_ = ~1.5 kbar (fig. S5). This transition is fully reversible with no sign of protein precipitation. Analysis of the pressure effects on the core residues of RRM2 used for the Go-model (fig. S4G) simulations showed that there was no correlation between these residues and the pressure effects. Meanwhile, comparison of the pressure data with the relaxation dispersion profiles for RRM2 (fig. S4H) did not reveal a notable correlation between the CPMG data and the pressure effects. We could not perform similar analysis for RRM1 as its residues did not display any significant change in intensity versus pressure.

These three sets of experiments demonstrate that: (i) despite their structural similarities, the isolated RRM2 is significantly less stable under pressure than the isolated RRM1, and (ii) RRM2 is stabilized when linked to RRM1 in the full UP1 domain. To investigate the origin of the stabilization of RRM2 in the UP1 domain, we first analyzed a variant where we swapped the position of the RRM domains (UP1^swap^; fig. S1B). Prediction by AlphaFold 2 (*21*) shows that the interface between the two RRMs and their relative orientation are substantially modified in UP1^swap^ compared to the WT UP1 (fig. S6). When pressurized, UP1^swap^ appears to be much less stable than WT UP1 (fig. S7, A, B, and E). Analysis of intensity profiles reveals two types of pressure sensitivity: a group of residues located within RRM2 that shows an almost complete loss of intensity at 2.5 kbar and a group of residues mainly located within RRM1 that exhibits only minor changes as a function of pressure (Fig. 3C).

The differential pressure-induced unfolding of UP1^swap^ and UP1 indicates that the integrity of the inter-RRM interface is a determinant of the thermodynamic stability and coupling of tandem RRMs of hnRNP A1. As a test of this concept, we examined the stability under pressure of a double salt bridge (R75D/R88D) variant of UP1 (UP1^dm^) (fig. S1B). This variant was designed to disrupt the salt bridges (R75:D155 and R88:D157) found at the interface between the two RRMs (insert of Fig. 1B). We previously showed that UP1^dm^ binds cognate RNA substrates with significantly weaker affinity compared to WT UP1 and that the salt bridge interactions stabilize the relative orientation of the tandem RRMs during a short 10-ns molecular dynamic simulation (*7*). Notably, Fig. 3D shows a similar clustering between pressure-sensitive and pressure-resistant residues for UP1^dm^ as observed for UP1^swap^. Two distinct groups of residues were also observed for UP1^dm^ based on intensity profiles: a group that shows almost complete intensity loss at 2.5 kbar (located within RRM2) and a group that displays only minor effect of pressure (mainly located within RRM1) (fig. S7, C, D, and F, and Fig. 3D). These results demonstrate that the integrity of the interface between the tandem RRMs plays a crucial role in stabilizing RRM2 in the context of the UP1 domain. Modifying the interface, as in UP1^swap^, or disrupting key interactions between the RRMs, as in UP1^dm^, disrupts the thermodynamic (allosteric) coupling between RRM1 and RRM2 (Fig. 3F).

Last, we sought to determine what factors contribute to the intrinsic lower thermodynamic stability of RRM2 compared to RRM1. We designed a variant of UP1 for which the sequence of RRM1 β strands are replaced by those of RRM2 (and vice versa) while keeping the interface between the two RRMs mostly intact (UP1^beta-trans^; fig. S1B). We observed for this variant a group of residues with high sensitivity to pressure, mainly corresponding to core residues within the modified RRM1, while a second group of residues, mainly within the modified core of RRM2, shows a much lower sensitivity to pressure (fig. S7, G and H, and Fig. 3, E and F). As described above, we identified both the extent of hydrophobic patches and packing density as the main structural differences between RRM1 and RRM2. Notably, the present results suggest that modifying these structural properties by transposing the sequences of β1-4 reverses the relative thermodynamic stability of RRM1 and RRM2.

### High-pressure SAXS reveals evidence of communication between the tandem RRMs and the low-complexity CTD

**Fig. 4.**
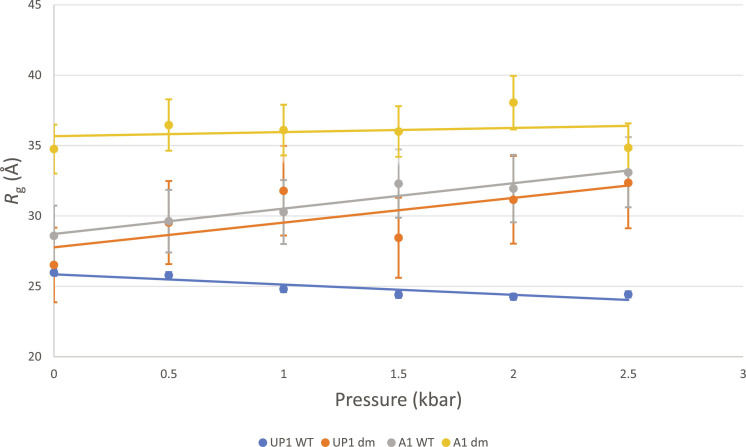
High-pressure SAXS studies of UP1 and full-length hnRNP A1. Numerical *R*_g_ values for UP1 and hnRNP A1 variants plotted versus pressure. *R*_g_ values were calculated from Guinier plots using BioXTAS RAW 2.1.4 (*55*).

High-pressure SAXS experiments were performed with four variants of hnRNP A1 to complement the NMR experiments described above: WT UP1, UP1^dm^, WT full-length hnRNP A1, and full-length hnRNP A1 R75D/R88D (A1^dm^). We tested each variant at separate pressures from 0 to 2.5 kbar, in increments of 500 bar, while monitoring the radius of gyration (*R*_g_) for each variant at each pressure (Fig. 4 and fig. S8, A to D). Results for WT UP1 analysis of the *R*_g_ values confirmed that the protein is stable and does not unfold under pressure. The *R*_g_ value measured for UP1^dm^ at atmospheric pressure is identical to that of the WT UP1, indicating no effect from the loss of the salt bridges on the overall globular structure of the protein. Yet, a sharp increase in *R*_g_ was observed under pressure for UP1^dm^, confirming, in excellent agreement with the NMR data, the loss of stability due to the loss of the inter-RRM salt bridges.

Next, we examined the effect of the double mutation R75D/R88D on the pressure stability of full-length hnRNP A1. We observed a slight increase in *R*_g_ values with increasing pressure for WT hnRNP A1, likely due to the low complexity C-terminal domain elongating from a compact to an extended conformation (fig. S8C), as previously reported (*22*). For A1^dm^, we found the *R*_g_ measured at atmospheric pressure to be significantly larger than that of the WT hnRNP A1. A similar result was also observed by size exclusion chromatography (SEC)–SAXS (fig. S8E), where *R*_g_ values obtained for WT hnRNP A1 (29.4 ± 0.23 Å and 28.7 ± 0.1 Å) were less than those obtained for A1^dm^ (33.9 ± 0.59 Å and 36.6 ± 0.71 Å), indicating that the WT is more compact than the mutant. As the UP1 domains are identical in size for both WT and mutant, such increase is likely due to an elongation of the C terminus (fig. S8D) that is affected by the loss of the R75:D155 and R88:D157 salt bridge interactions that stabilize the inter-RRM interface in the UP1 domain. Application of pressure reveals a further increase in *R*_g_ values measured for A1^dm^, suggesting a complete unfolding of the UP1 domain (Fig. 4). These results confirm that the salt bridge disrupting mutations markedly affect the stability under pressure of the tandem RRMs, for both the full-length hnRNP A1 and isolated UP1.

### Molecular dynamic simulations reveal that the inter-RRM’s integrity influences the low complexity CTD

We performed molecular dynamic simulations to assess how the domains of hnRNP A1 interact with each other and how the R75D/R88D mutations disrupt these interactions. For WT UP1, the interactions of the tandem RRM domains were mapped out (fig. S9, A and B). The simulations revealed an interaction surface centered around the salt bridge interactions but also encompassing the flexible N terminus of RRM1, α_1_ on RRM2, and the loop between α_2_ and β_4_ on RRM2. These results present the possibility of RRM2 positioning the N terminus and its 3_10_ helix for formation of the RNA binding pocket it forms with the inter-RRM linker (*7*). Identical simulations for UP1^dm^ revealed almost all inter-RRM interactions are ablated (fig. S9, C and D). With the loss of the salt bridge interaction, RRM2 loses its interactions with the RRM1 N terminus and cannot position it for formation of the RNA binding pocket with the inter-RRM linker, confirming previous simulations (*7*).

For simulations with full-length hnRNP A1, we divided the protein into four domains: RRM1 (1 to 90), inter-RRM linker (91 to 106), RRM2 (107 to 182), and LCD_A1_ (183 to 320). The interactions for LCD_A1_ with each other domain was then analyzed. The simulations revealed that LCD_A1_ shares a narrow interaction surface with the inter-RRM interface formed between the N terminus of RRM1 and the α_2_/β_4_ loop of RRM2 (fig. S9, E to G). Further contact with the tandem RRM domains was found with the β_2_/β_3_ loop of RRM1 and β_4_ of RRM2. With A1^dm^, the binding surface between RRM1 and LCD_A1_ was left primarily intact, except the RRM1 N terminus (fig. S9, I and J). For RRM2 (fig. S9K), the loss of the salt bridges ablated all interactions between RRM2 and LCD_A1_, and only a single interaction between K183 and E135 was detected. This result demonstrates that the allosteric coupling that exists between the tandem RRMs on hnRNP A1 propagates to its C-terminal domain.

For the inter-RRM linker, the interactions with LCD_A1_ were largely identical between hnRNP A1 (fig. S9H) and A1^dm^ (fig. S9L), with one notable difference. In hnRNP A1, the linker interacts with the RGG box region of LCD_A1_, whereas in A1^dm^, the RGG box residues shift from interacting with the linker to interacting with RRM1. They interact with two different regions of RRM1, residues 24 to 27, in the α helix between β_1_ and β_2_, and residues 47 to 55, in the loop between β_2_ and β_3_.

### Thermodynamic coupling of the tandem RRMs of hnRNP A1 regulates its RNA binding ability and multimerization

**Fig. 5.**
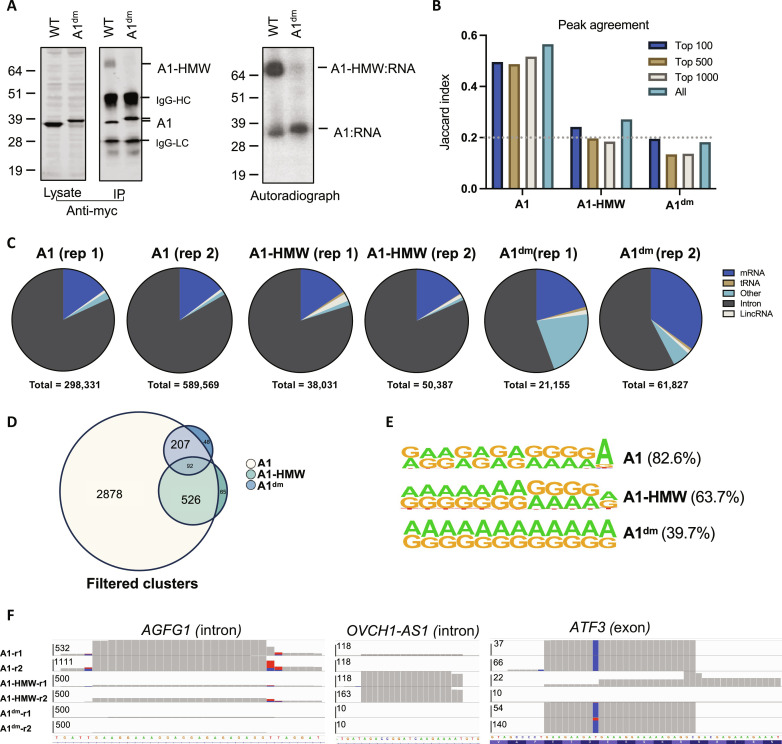
PAR-CLIP analysis reveals distinct RNA binding profiles of WT hnRNP A1 and the A1^dm^ mutant. HEK293T cells transfected with myc-tagged WT hnRNP A1 and the A1^dm^ mutant were processed for PAR-CLIP. (**A**) Cell lysates and immunoprecipitated protein-RNA complexes were analyzed by immunoblotting using an anti–hnRNP A1 antibody or autoradiography. A1-HMW denotes the high molecular weight hnRNP A1 variant. HC and LC denote the heavy and light chain of the immunoprecipitating antibody. (**B**) Bar plot showing the Jaccard similarity index of the peaks identified in replicate experiments. (**C**) Number of PAR-CLIP reads that map to the indicated RNA species within the peaks (clusters) from the two independent PAR-CLIP libraries are shown. (**D**) PAR-CLIP–derived peaks for each hnRNP A1 variant were filtered to remove those with less than 10 reads and were present only in one replicate. Euler diagram shows the overlap of the identity of filtered peaks (not taking into consideration how frequently each peak is bound) across WT hnRNP A1, A1-HMW, and A1^dm^. (**E**) Motif analysis within the filtered peaks bound by monomeric WT hnRNP A1, A1- HMW, and the A1^dm^ mutant. Percentage of clusters containing the indicated motifs is shown in parantheses. (**F**) Exemplary Integrated Genomics Viewer PAR-CLIP traces on the indicated genes are shown. Numbers on *y* axes indicate mapped read counts. IgG, immunoglobulin G; lincRNA, long intergenic noncoding RNA.

To determine how the observed thermodynamic coupling of the tandem RRMs of hnRNP A1 affects its RNA binding properties in a physiologically relevant setting, we next conducted photoactivatable ribonucleoside-enhanced cross-linking immunoprecipitation (PAR-CLIP) studies as described before (*23*, *24*) for WT hnRNP A1 and the A1^dm^ variant bearing the R75D/R88D salt bridge substitutions within the UP1 domain. To this end, human embryonic kidney (HEK) 293T cells were transiently transfected with constructs expressing myc-tagged WT hnRNP A1 and A1^dm^ and subsequently grown in the presence of a photoactivatable ribonucleoside analog, 4-thiouridine, which gets incorporated into nascent RNA. Cells were ultraviolet (UV)–cross-linked, and A1/A1^dm^-RNA complexes were immunoprecipitated from lysates. Both WT hnRNP A1 and A1^dm^ proteins were expressed well in cells and immunoprecipitated efficiently (Fig. 5A). For WT hnRNP A1 but not the A1^dm^ variant, we noted the presence of an additional high molecular weight (A1-HMW) band in the immunoprecipitated material that was significantly less abundant than the monomeric protein (Fig. 5A). Of note, A1-HMW migrated at ~70 kDa, possibly corresponding to a dimeric hnRNP A1. We found that hnRNP A1-HMW immunoprecipitated in complex with proportionally higher RNA levels than monomeric hnRNP A1 or the A1^dm^ variant (Fig. 5A).

In two independent PAR-CLIP experiments, bound RNA was further purified away from monomeric hnRNP A1, A1-HMW, as well as monomeric A1^dm^ and sequenced. Reassuringly, PAR-CLIP–derived reads in all six libraries predominantly contained T-to-C substitutions when mapped to the reference genome (fig. S10A), a result of mutations introduced at the protein-RNA cross-linking sites containing 4-Thiouridine (4-SU) during the reverse transcription step of library generation. Reads that uniquely map to the human genome were then clustered to identify the peak binding sites for each variant. Peak agreement between replicate samples, as indicated by Jaccard index, was within an acceptable range (0.2 to 0.4) expected from CLIP experiments (Fig. 5B). Of the over 10,000 peaks for monomeric hnRNP A1 (fig. S10B and table S1), ~90% of the peaks were located within the introns, a finding in agreement with prior PAR-CLIP studies (*25*). PAR-CLIP with A1-HMW and A1^dm^ yielded an average of 3000 and 2000 peaks, respectively (fig. S10B and table S1). Comparative analysis of binding site occupancies across all binding sites show distinct variations. For example, while the A1-HMW variant showed enriched binding to introns similar to the monomeric hnRNP A1, the A1^dm^ mutant more frequently associated with mRNAs and other RNA species [i.e., noncoding RNAs, small nuclear RNAs, miscellaneous RNAs (miscRNAs), etc.] (Fig. 5C, fig. S10B, and table S1). More stringent analysis of binding sites by filtering out peaks with low read counts (<10) and are present only in one replicate experiment yielded a similar result (fig. S10C). This analysis also revealed that the PAR-CLIP–derived peaks for both A1-HMW and A1^dm^ were largely a subset of A1-derived peaks but minimally overlapped with each other (Fig. 5D and table S1). Despite these differences, consensus motif sequences identified by PAR-CLIP for hnRNP A1, A1-HMW, and the A1^dm^ variant were similar and AG-rich (Fig. 5E). Exemplary CLIP traces provide additional support. For example, the highly purine-rich *AGFG1* (intron) is bound by A1, less frequently by A1-HMW, and not bound by A1^dm^; *OVCH1-AS1* (intron) is only bound by A1-HMW; *ATF3* (exon) is bound most frequently by A1^dm^ and not by A1-HMW (Fig. 5F). Together, the difference in binding capacity between WT hnRNP A1 and the A1^dm^ mutant correlated with the latter being defective in its ability to dimerize on the immunoprecipitated transcripts.

## DISCUSSION

### HnRNP A1 is an allosterically regulated RNA binding protein

Knowledge as to how hnRNP A1 converts recognition of short degenerate RNA sequence motifs, ubiquitous across a transcriptome, into regulated biological outcomes remains enigmatic. Numerous atomic-resolution structures and high-throughput binding studies offer insights into the mechanisms by which hnRNP A1 achieves specificity for a minimal 5′-YAG-3′ motif (*1*, *26*); however, these cumulative observations provide only limited understanding into the processes by which hnRNP A1 modulates general RNA metabolism. This study provides evidence that hnRNP A1 is in part intrinsically regulated via the thermodynamic coupling of its tandem RRM domains. The significance of intrinsic regulation is that hnRNP A1’s RNA binding capacity can be tuned by long-range communication between its tandem RNA Recognition Motifs (RRMS) and its LCD, naturally occurring mutations, and PTMs (*2*).

In this study, we demonstrate that thermodynamic coupling of the tandem RRMs of hnRNP A1 regulates its RNA binding capacity and likely its ability to dimerize. Since specific RNA binding and dimerization are partitioned to the N-terminal UP1 domain and the LCD_A1_, respectively, we suggest that thermodynamic coupling of the tandem RRMs is the foundational basis of allosteric regulation of hnRNP A1. On the basis of the previously determined UP1-(AGU) crystal structure, we built a data-driven structural model of UP1 bound to HIV-1 SL3^ESS3^, a 25-nucleotide stem loop with a high-affinity UAG binding site in its apical heptaloop (*7*). In the model, the center of the β sheet surface of RRM2 is more than 20 Å away from the heptaloop surface. The observation of this univalent binding mechanism opened the possibility that hnRNP A1 is an allosterically regulated RNA binding protein. Of further support of this concept, we demonstrated that mutating the R75:D155/R88:D157 salt bridge interactions, which stabilize the inter-RRM interface, was sufficient to reduce the affinity for SL3^ESS3^ by more than 18-fold with an accompanied decrease in total binding enthalpy (*7*). While the large reduction in binding affinity alone does not satisfy conditions for allostery, the comparative high-pressure NMR studies of WT UP1 and UP1^dm^ determined here clearly show that the integrity of the inter-RRM interface is necessary to impart uniform thermodynamic stability across the surface of the N-terminal domain of hnRNP A1. Decoupling the interface either via salt bridge mutations or RRM transposition exposes the significantly less stable RRM2 domain. Our results further revealed that the differential thermodynamic stabilities and conformational dynamics of RRM1 and RRM2 correlate with the extent of hydrophobic packing. Thus, this work demonstrates that the physicochemical basis of hnRNP A1 allostery is thermodynamic coupling, which acts to normalize the stabilities of the RRMs and to provide an intermolecular network to communicate binding site occupancy.

Allosteric regulation of hnRNP A1 offers a mechanistic rational to interpret its pleiotropic RNA binding functions. LCD_A1_ participates in heterotypic protein-protein and protein-RNA interactions, where the extent to which such interactions form influences the functional behavior of hnRNP A1. The RNA-processing regulatory functions of hnRNP A1 require it to dimerize or oligomerize along the nascent transcripts. Studies to determine the mechanisms that hnRNP A1 uses for splicing repression revealed two possible RNA-dependent mechanisms. The protein can dimerize while bound to distal regulatory sites to “loop out” the RNA, or it can oligomerize along the RNA to sterically block spliceosome assembly (*27*, *28*). These mechansims reveal how allosteric coupling of the tandem RRMs and the LCD_A1_ allows fine-tuning of hnRNP A1’s heterotypic interactions via its specific affinity for RNA substrates that contain optimal or near optimal consensus motifs (*26*).

Related to this concept, the Mittag group (*29*) found that the LCD_A1_ lays over the tandem RRMs. This interaction is electrostatically driven and dependent on the salt concentration of the solution buffer. This interaction drove stress granule assembly, as a lower salt buffer concentration led to greater interdomain interaction and greater granule assembly. More recently, the Jeschke group performed Paramagnetic Relaxation Enhancement (PRE) experiments with labeled residues in the LCD_A1_ to determine the points of contact between the two domains (*30*). They placed one such probe at residue 231 in the RGG box region. This probe showed clusters in elements important for RNA recognition such as the inter-RRM linker and the β2-β3 loop of RRM1. Since LCD_A1_ occupies the same space as the RNA, they hypothesized that the RNA would displace LCD_A1_ from its binding surface. Our molecular dynamic simulations revealed similar results, as we found LCD_A1_ interacts with the inter-RRM linker and the RRM1 N terminus, two elements that act in tandem to form a part of the RNA binding pocket. Our simulations also revealed that the RGG box residues interact with the linker in WT hnRNP A1 but switches to interacting with the β_2_-β_3_ loop in the A1^dm^ mutant.

The examination of phase separation by hnRNP A1 revealed a regulatory and concentration-dependent link between RNA binding by the UP1 domain and the rate of stress granule assembly induced in LCD_A1_ for hnRNP A1 (*31*–*33*). This observed behavior from the protein implies a communicative link between the RNA binding domains and the disordered C-terminal domain, with regulatory signals being transmitted either sequentially, or through “layover” structural interactions. The UP1-(AGU) crystal structure revealed that RRM1 is capable of binding RNA via a univalent mechanism; thus, any sequential signaling to LCD_A1_ would be disrupted by the salt bridge mutations of A1^dm^. The RNA-dependent dimerization observed in the PAR-CLIP experiments provides further evidence for this signaling link between RNA binding in UP1 and complex formation in LCD_A1_.

The previous research mentioned above, along with the data presented here, reveals that hnRNP A1 acts as a single functional unit through allosteric regulation. While each domain has its particular function, changes in one domain can be transmitted to others to affect their function. This is seen in the analysis of the PTMs of hnRNP A1 (Table 1). The majority of the PTMs occur in either RRM1 or LCD_A1_ and affect the function of the specific domain of its location; RNA binding for RRM1 and complex formation for LCD_A1_. A few of these PTMs affect the function of the other domains. The ubiquitination of K183 confirms the regulatory role of RRM2 in RNA binding (*34*). The modification at this residue was shown to disrupt the RNA-protein interaction by conformational changes in RRM2. In LCD_A1_, the methylation of Arg^218^ and Arg^225^ decreases the ability of hnRNP A1 to activate internal ribosomal entry site–mediated translation while also reducing its IRES trans-acting factors (ITAF) activity (*35*–*37*). This regulation of identity-specific RNA binding activity could occur by disrupting functionally specific complex formation required to direct hnRNP A1 to its RNA substrate. This RNA complex–dependent regulation could explain our observation through PAR-CLIP that the A1^dm^ mutant, which loses interdomain coupling, binds RNA of different identity.

**Table 1.** PTMs of hnRNP A1. Compilation of PTMs made to hnRNP A1 and their locations on the protein. [Adapted from (*2*)].

Modification	RRM1	RRM2	LCD
Phosphorylation	Serine 4 (*66*)	Serine 192 (*67*)	Serine 199 (*68*)
	Serine 6 (*66*)		Serine 310–312 (*67*)
	Threonine 51 (*41*)		
	Serine 95 (*69*)		
Methylation	Arginine 31 (*70*)		Arginine 206 (*37*)
			Arginine 218 (*35*)
			Arginine 225 (*36*)
			Arginine 232 (*37*)
Ubiquitination	Lysine 3 (*71*)	Lysine 183 (*34*)	Lysine 298
	Lysine 8 (*71*)		
	Lysine 15 (*71*)		
Acetylation	Lysine 3 (*72*)		
	Lysine 52 (*72*)		
	Lysine 87 (*72*)		
SUMOylation		Lysine 183 (*66*)	
β-*N*-acetyl-glucosamine-ylation	Serine 22 (*73*)		
PARylation			Lysine 298 (*74*)

**Fig. 6.**
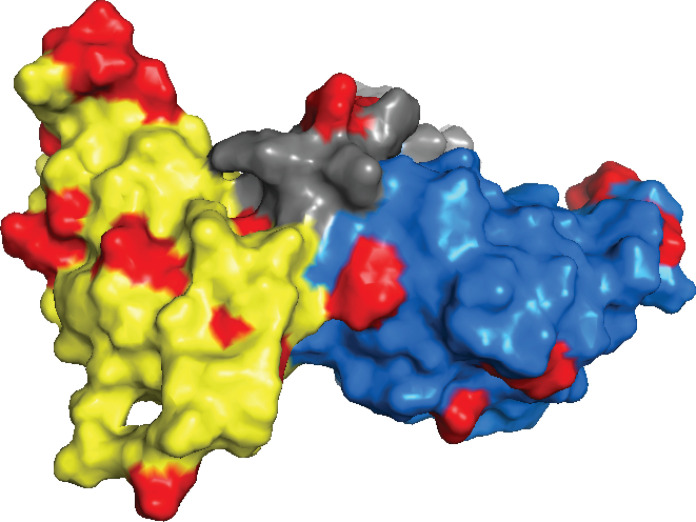
Location of cancer-associated amino acid variations. Surface representation of cancer-associated mutations in UP1 (PDB 2lyv) compiled from the Cancer Cell Line Encyclopedia (D27N, D27E, E28K, P49S, N50D, R53H, G56S, T61A, V84L, P98L, V109I, D123N, H156R, V159L, K161E, Q165L, E185Q, and S191C) and The Cancer Genome Atlas (S4L, E9R, D42N, R55M, A89V, Q96R, R140P, S142I, K161E, N171D, and V177A). RRM1 is colored yellow, RRM2 is colored blue, and the linker between the two RRMs is colored gray. Residues identified in this compilation are colored red.

The analysis of PTMs revealed how alterations to one amino acid can cause global changes to protein function or stabilization. The identification of single amino acid variations (SAVs) has shown similar effects. Many of these SAVs cause protein destabilization and have been linked to cancer (*38*–*40*). We conducted searches for SAVs in hnRNP A1 using cancer-linked mutational databases built from somatic cells (Cancer Genome Atlas) and germline cells (Cancer Cell Line Encyclopedia). This survey revealed more than 30 potential cancer-associated mutations (~15% of UP1 sequence) map to the tandem RRMs of hnRNP A1 with several having the potential to disrupt the thermodynamic coupling described here (Fig. 6).

One of the major questions presented by our findings is what specific advantage does hnRNP A1 gain from having a stable RRM1 appended to an unstable RRM2. The stability of hnRNP A1 is important physiologically, as the protein’s stability plays a role in cellular survival. Previous studies revealed that as a response to endoplasmic reticulum stress, eukaryotic initiation translation factor 3 alpha kinase 3 (Protein Kinase R-like ER Kinase, PERK) phosphorylates Thr^51^ on RRM1, leading to its degradation (*41*). This degradation occurs with its unfolding, potentially aided by its coupling to the unstable RRM2 domain.

When combining these various observations, it becomes more likely that allostery intrinsically underlies the regulatory capacity of hnRNP A1 to engage with its various RNA targets. Allostery is a well-established property of multidomain enzymes, which coordinate multiple biomolecular inputs to catalyze functional outcomes (*18*). Enzymes that function in primary metabolism are almost universally allosterically controlled by substrate binding and PTMs. Efforts to identify and characterize potential allosteric control of RNA binding proteins that recognize short degenerate sequences might offer critical insights into RNA biology. Further, this work demonstrates how allostery can be masked via thermodynamic coupling of domains and the importance of using various biophysical methods to elucidate it.

## MATERIALS AND METHODS

### Constructs and protein purification

All protein constructs were cloned from gBlock gene fragments (Integrated DNA Technologies, IDT) into pMCSG plasmid (*42*). UP1 and RRM constructs were cloned with an N-terminal 22–amino acid His-Tag, while full-length hnRNP A1 constructs were cloned with an N-terminal GB1 solubility domain with a His-Tag. All constructs contained TEV cleavage sites for removal of the His-Tag or GB1 domain.

Protein was overexpressed as previously described (*22*). UP1 and RRM proteins were purified as previously described (*43*, *44*). Full-length hnRNP A1 was purified in similar manner but with lysis buffer composition of 20 mM Na_2_HPO_4_ (pH 7.5), 1.2 M NaCl, 0.5 mM EDTA, 2 M urea, 20 mM imidazole, and 1 mM phenylmethylsulfonyl fluoride. Elution buffer composition was identical except imidazole concentration was 250 mM. Fast protein liquid chromatography gel filtration buffer was 100 mM Hepes (pH 7.5) and 1 M NaCl. Salt concentration was decreased by serial dilutions with 100 mM Hepes (pH 7.5) while simultaneously concentrating protein sample through Amicon filtration.

### Go-model simulations

List of native contacts for the all-atom simulations with a structure-based potential (Go-model) was established with SMOG algorithm (*45*) using the coordinates of RRM1 and RRM2 extracted from PDB 1U1R as a reference structure. All-atom simulations were performed in GROMACS 2018.8 using the leapfrog integration method with 2-fs time steps (*46*). For each RRM, independent simulations were performed over a wide range of temperatures, and the folding temperature (*T*_f_) was determined with the weighted histogram analysis method (*47*). Analysis of the trajectories was carried out using in-house Python scripts.

### High-pressure NMR

All NMR spectra were acquired on a Bruker 700 spectrometer equipped with z-shielded gradient triple resonance 5-mm TCI cryoprobe. Experiments were run with the proteins at concentrations of ~250 μM in buffer consisting of 100 mM Hepes (pH 7.5), 350 mM NaCl, 0.5 mM EDTA, and 10% ^2^H_2_O. Hydrostatic pressure was controlled using a commercial ceramic high-pressure NMR cell and an automatic pump system (Daedalus Innovations, Philadelphia, PA). 2D ^1^H-^15^N HSQC experiments were recorded at 290 K with a time domain matrix consisting of 100* (*t*_1_, ^15^N) × 1024* (*t*_2_, ^1^H) complex points with acquisition time of 50 ms (*t*_1_) and 91.8 ms (*t*_2_) using 1.5-s interscan delay. 2D ^1^H-^15^N spectra were collected every 250 bar from 1 bar to 2.5 kbar using 20-min equilibration delay after every change of pressure. All spectra were processed using NMRPipe (*48*) and displayed with SPARKY (*49*).

### Relaxation dispersion

NMR relaxation dispersion experiments were acquired on a 600-MHz Bruker magnet equipped with a 5-mm TXI cryoprobe at 298 K. Experiments were run with the proteins at concentrations of ~350 μM in buffer consisting of 100 mM Hepes (pH 7.5), 350 mM NaCl, 0.5 mM EDTA, and 10% ^2^H_2_O. Amide ^15^N CPMG experiments were acquired using the single-train continuous wave (STCW)-CPMG (*50*) pulse sequences and recorded in a pseudo-3D fashion. The constant relaxation time was set to 40 ms, and the CPMG pulsing frequency, ν_CPMG_, was varied from 25 Hz to 1.5 kHz. To prevent spectral artifacts arising from sample instability, the order of the acquisition of increments along the indirect *t*_1_ dimension was randomized and interleaved with the number of scans, repeating the minimal phase cycle four times. NMR data were processed using nmrPipe (*48*), and intensities were extracted using the autoFit routine in the NMRPipe suite. Errors in signal amplitudes were estimated from two to three replicate ν_CPMG_ measurements for each CPMG experiment and used to propagate the errors in *R*_2,eff_, where

$$R_{2,\text{eff}}=-\frac{\text{ln}\left[\frac{I\left(\nu_{\text{CPMG}}\right)}{I_{0}}\right]}{T_{\text{rlx}}}$$

*I*_0_ is the signal amplitude without the CPMG element, *I*(ν_CPMG_) is the amplitude at the given ν_CPMG_, and *T*_rlx_ is the constant relaxation time. The median errors in *R*_2,eff_ were 0.4, 1.6, and 3.3 s^−1^ for ^15^N datasets of RRM1, RRM2, and UP1, respectively. The ^15^N CPMG profiles were analyzed to identify those residues with *R*_ex_ = *R*_2,eff_[min(ν_CPMG_)] − *R*_2,eff_[max[ν_CPMG_)] greater than 1.65 times the *R*_ex_ measurement error, σ_*R*ex_, amounting to 95% confidence for the presence of exchange. Those residues identified in this fashion were then fit to simple analytical models of fast and slow exchange (*51*) to estimate the initial two-state exchange parameters, the total rate *k*_ex_ = *k*_ab_ + *k*_ba_, and population *p*_b_. All exchange was in the fast regime with *k*_ex_ on the order of ~200 to 2000/s with the most accurate results for RRM1 of about 175/s. Populations of the excited state could not be obtained. Processed spectra were displayed with SPARKY (*49*).

### High-pressure SAXS

The HP-SAXS data were collected at the sector 7A1 station (HP-Bio) of the Cornell High Energy Synchrotron Source (CHESS) (*52*–*54*). Pressure was maintained by a Barocycler HUB440 high-pressure pump (Pressure BioSciences). Samples were illuminated with a 250 μm by 250 μm x-ray beam of wavelength λ = 0.8823 Å (14.05 keV), with a flux of ∼2.1 × 10^11^ photons/s. Scattering was measured with an EIGER 4M detector (Dectris) in vacuum with a pixel size of 75 μm × 75 μm and an active area of 155.1 mm by 162.2 mm (2068 × 2162 pixels), over the *q* range of ∼0.01 to 0.6 Å^−1^ [where *q* is the wave vector defined as *q* = (4π sinθ/λ) and 2θ is the scattering angle].

Samples were prepared in 100 mM Hepes (pH 7.5), 350 mM NaCl, and 0.5 mM EDTA at 5 (full-length hnRNP A1 samples) and 10 (UP1 samples) mg/ml. HP-SAXS measurements were carried on from 1 bar to 2.5 kbar at 290 K with 10 exposures of 1.0 s each (for a total of 10-s exposure at each dataset). The 2D SAXS images were azimuthally integrated about the beam center and normalized by the transmitted intensities via standard image correction procedures using the BioXTAS RAW 2.1.4 software package (*55*). Data were placed on an absolute scale using water as a standard. The specimen-to-detector distance was set to 1804.0 mm. Data analysis was performed with BioXTAS RAW 2.1.4 (*55*), with utilization of DAMMIF (*56*). SAXS envelopes were constructed and visualized using Chimera (*57*).

### Photoactivatable ribonucleoside-enhanced cross-linking immunoprecipitation

HEK293T cells were grown in Dulbecco’s modified Eagle’s medium supplemented with 10% fetal bovine serum. HEK293T were seeded in 10-cm dishes and then transfected with 10 μg of myc-tagged hnRNPA1 and hnRNP A1^dm^ mutant using polyethyleneimine (PolySciences). Cells were treated with 100 μM 4-SU 16 hours before UV–cross-linking. PAR-CLIP was performed as previously described (*24*). Briefly, cells were lysed in 1× radioimmunoprecipitation assay buffer supplemented with protease inhibitors. hnRNP A1/A1^dm^-RNA complexes were immunoprecipitated by a rabbit polyclonal antibody against the myc tag (Thermo Fisher Scientific, #PA1-981) and the bound RNA molecules end-labeled with T4 polynucleotide kinase (PNK) and γ-^32^P-ATP. Protein-RNA complexes were resolved on 4 to 12% NuPAGE SDS–polyacrylamide gel electrophoresis gels, transferred to nitrocellulose membranes, and visualized by autoradiography. Bound RNA was further purified away from protein-RNA complexes by proteinase K treatment and processed by sequential ligation of 3′ and 5′ adapters. Resulting libraries were subjected to reverse transcription polymerase chain reaction and sequenced on an Illumina NextSeq instrument for 75 cycles.

### Analysis of PAR-CLIP data

PAR-CLIP–derived reads were first processed using BBduk to remove adapter sequences and separated into barcodes, removing reads of length less than 10 nucleotides. The resulting reads were filtered off of ribosomal RNA mapped to the human transcriptome using STAR aligner, allowing mismatch in less than 10% of read length (*58*). Mapped reads were further analyzed by R package wavClusteR for the presence of T-to-C substitutions and by PARalyzer (*59*) to generate peaks (i.e., binding sites) and annotated with annotatePeaks.pl by HOMER (*60*). To calculate the peak agreement across replicates, we used the Jaccard distance metric using RCRUNCH as described in (*61*, *62*). Jaccard index was calculated separately for peaks ranked by read count (i.e., top 100, top 500, and top 1000 peaks with highest read counts) as well as all peaks. For certain subsequent analyses, peaks that are present only in one replicate experiment and contained less than 10 read counts were filtered out. Note that while some figures display the features of these clusters, other figures indicate the number of reads that map to each cluster as indicated in figure legends. Subsequently, the resulting peaks were subjected to motif analysis using HOMER (*60*), and overlap of peaks across hnRNP A1 variants was assessed by custom scripts.

### Molecular dynamic simulations

The full-length hnRNP A1 protein (1-320) was developed using AlphaFold (*21*). Coordinates for hnRNPA1 R75D/R88D mutant PDB were designed via PyMOL and based on the initial orientation of full-length hnrnp A1 (1-320). The protonation of the protein and the fine-tuning of the hydrogen bond network were facilitated through the Protein Prepare module in HTMD (*63*).

The systems were prepared for simulation via HTMD, with water padding configured at a distance equivalent to the furthest atom of the protein plus an extra 10 Å from the center. The system was neutralized with the addition of sodium and chloride ions (0.1 M). For systems, an equilibration process was implemented, followed by production cycles of 1 μs each per system.

The equilibration protocol comprised 500 steps each of minimization in NVT ensemble, followed by 1-ns in NPT ensemble. Heavy atoms were subjected to restraints of 1 kcal/mol, and nonheavy atoms had restraints of 0.1 kcal/mol, which were progressively lifted until midway through the simulation when the system became entirely free of restraints.

The production simulations ran without restraints for a duration of 1 μs. The CHARMM22* force field was used, with simulations executed on a cluster equipped with a single GPU, using ACEMD simulation software on NMRBOX (*64*). Using Bridg2, an analysis was conducted on the hydrogen bond network and its occupancy over the 1-μs trajectory (*65*). The objective was to uncover any potential interactions between the RRM domains, inter-RRM linker, and C-terminal domain within the established hydrogen bond network of WT hnRNP A1 and R75D/R88D mutant. Through the Bridg2’s built in tools, the hydrogen bond network graph and its occupancy were established on the basis of the residue numbers pertaining to the specific domains.
