# Supplementary material for: Thermodynamic coupling of the tandem RRM domains of hnRNP A1 underlie its pleiotropic RNA binding functions
Source: Sci Adv. 2024 Jul 10;10(28):eadk6580. doi: 10.1126/sciadv.adk6580 (PMC11235170; doi:10.1126/sciadv.adk6580)
Supplement: Supplementary file 1 — Figs. S1 to S10 Table S2 Legend for table S1 [file sciadv.adk6580_sm.pdf]

Science Advances
AAAS

Supplementary Materials for

Thermodynamic coupling of the tandem RRM domains of hnRNP A1 underlie its pleiotropic RNA binding functions

Jeffrey D. Levengood *et al.*

Corresponding author: Julien Roche, jroche@iastate.edu;
Blanton S. Tolbert, blanton.tolbert@pennmedicine.upenn.edu

Sci. Adv. **10**, eadk6580 (2024)
DOI: 10.1126/sciadv.adk6580

The PDF file includes:

Figs. S1 to S10
Table S2
Legend for table S1

Other Supplementary Material for this manuscript includes the following:

Table S1

A

```
RRM1 4    LRKLFIGGLSFETTDESLRSHFEQWGTLTDCVVMRDPNTKRSRGFGFVTYATVEEVDAAM  63
          ++K+F+GG+  +T +     LR  +FEQ+G +          +M  D + +      RGF   FVT+        + VD  +
RRM2 3    VKKIFVGGIKEDTEEHHLRDYFEQYGKI E V IEI  MTDRGSGKKRGFAFVTFDDHDSVDKIV  62

RRM1 64   NARPHKVDGRVVEPKRAVSR  83
             + H   V+G        E ++A+S+
RRM2 63   IQKYH TVNGHNCEVRKALSK  82
```

B

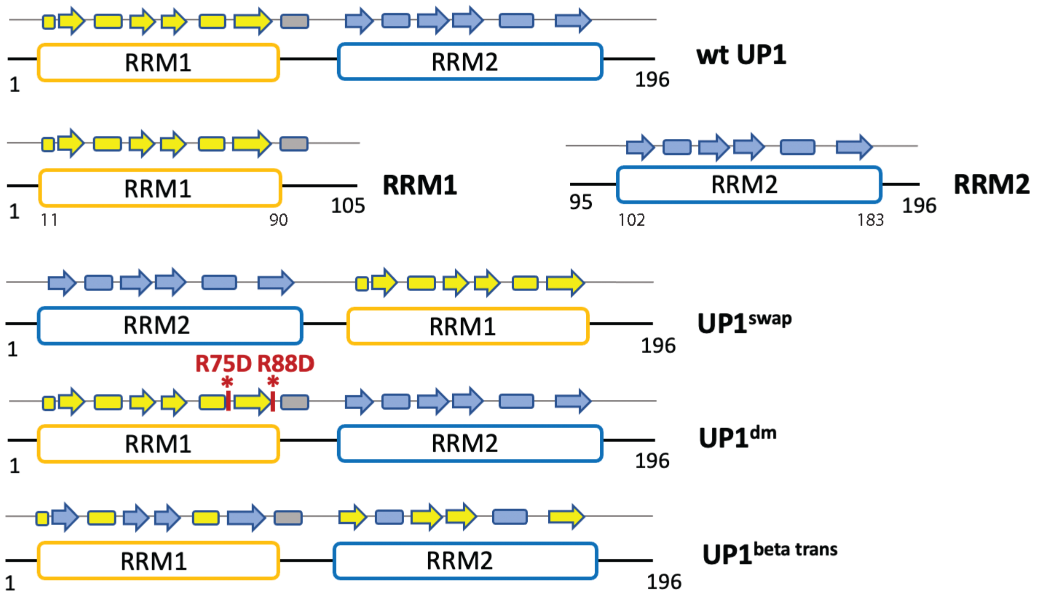

Figure S1. **Sequence and construct feaures of the RRM domains and UP1 variants.** (**A**) Sequence alignment of RRM1(11-90) and RRM2(102-183) carried out for the structural core regions of the two domains. Residues that match or share chemical properties are identified in the space between the two sequences. Underlined residues represent the 10 residues of the structural core of each domain with the lowest accessible surface area (**B**) List of constructs used in this study, including from top to bottom: (*i*) wt UP1 domain of hnRNP A1, (*ii*) the isolated RRM1 and RRM2 motifs, (*iii*) UP1^{swap} variant for which the RRM2 motif is positioned at the N-terminus and RRM1 at the C-terminus, (*iv*) UP1^{dm,} a construct bearing two mutations (R75D, R88D) designed to disrupt two salt bridges at the interface between the two RRMs, and (*v*) UP1$^{beta\text{-}trans}$ for which the sequences of the four β-strands are transposed between RRM1 and RRM2.

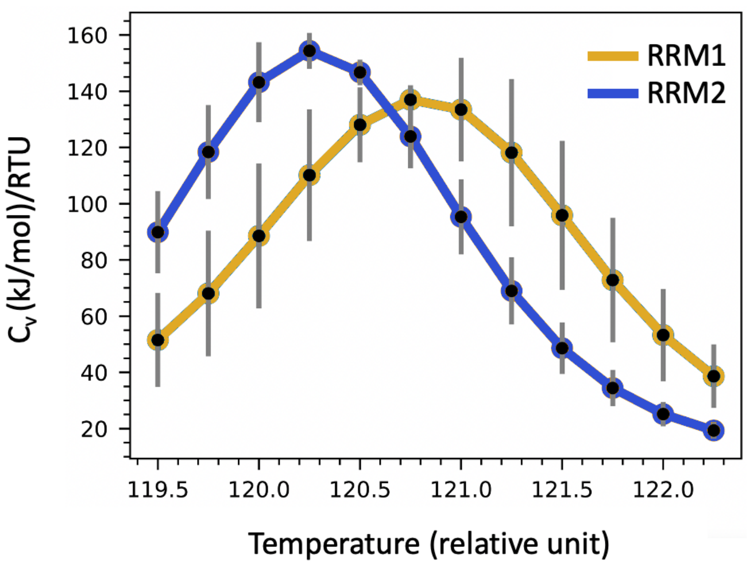

Figure S2. Thermodynamic stability of the RRM domains. Specific heat capacity measured from all-atom molecular simulations conducted on the individual RRMs with a structure-based (Go-model) potential. These simulations predict that the isolated RRM2 (blue) is slightly less thermodynamically stable than RRM1 (yellow).

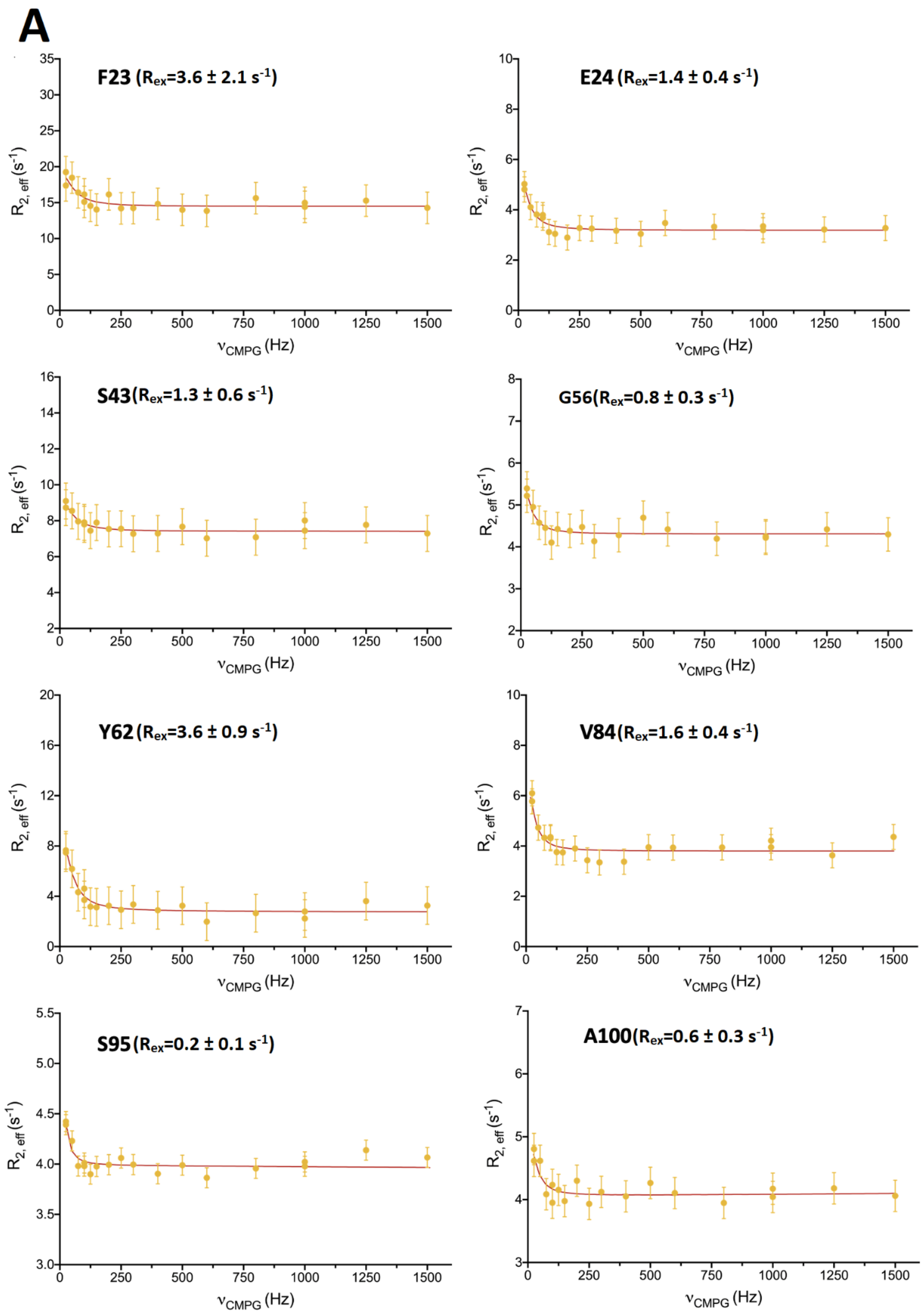
A
F23 (Rex=3.6 ± 2.1 s-1)
E24 (Rex=1.4 ± 0.4 s-1)
S43(Rex=1.3 ± 0.6 s-1)
G56(Rex=0.8 ± 0.3 s-1)
Y62(Rex=3.6 ± 0.9 s-1)
V84(Rex=1.6 ± 0.4 s-1)
S95(Rex=0.2 ± 0.1 s-1)
A100(Rex=0.6 ± 0.3 s-1)
R2, eff (s-1)
νCMPG (Hz)

B

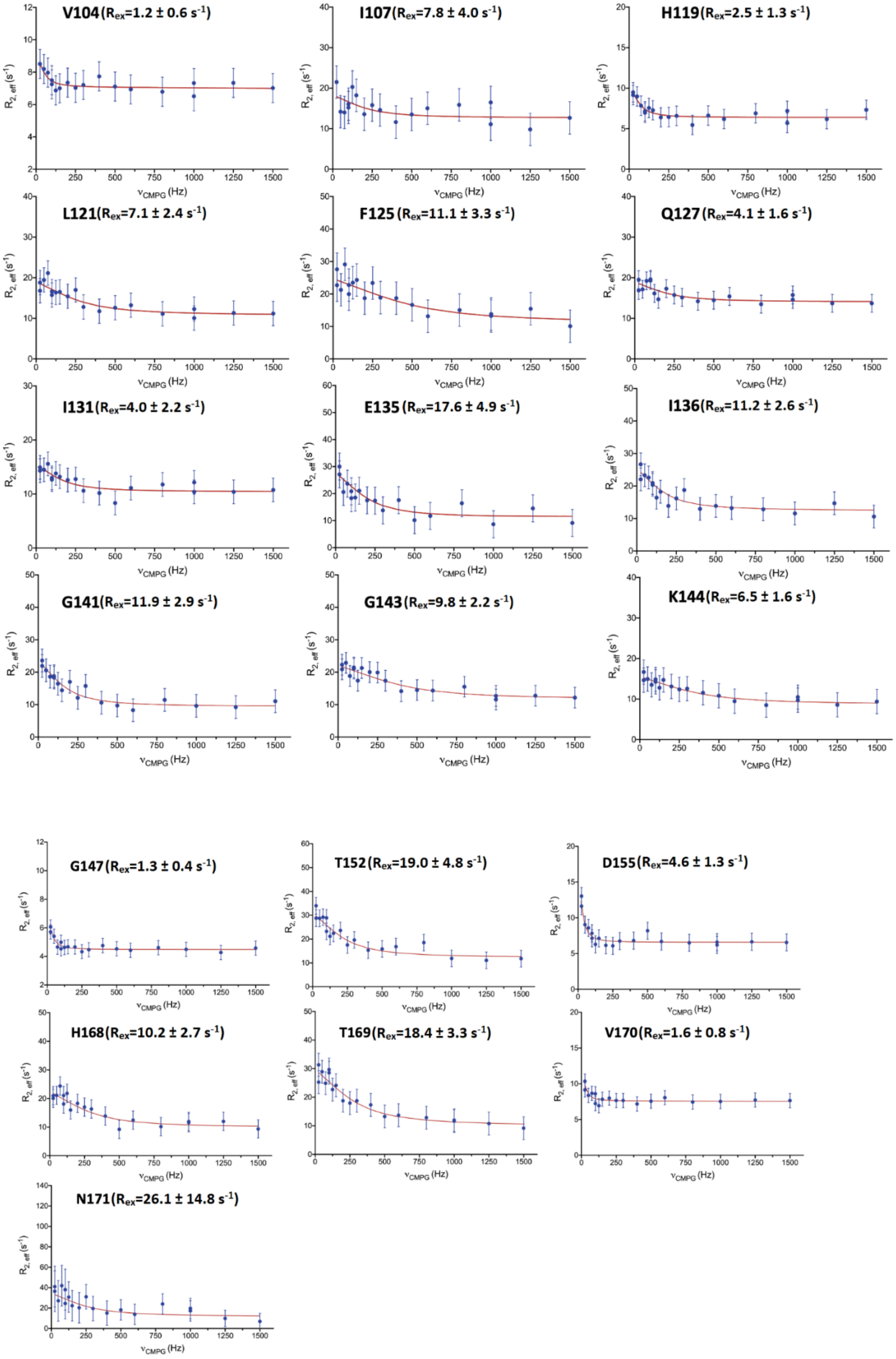

V104 (R_{ex}=1.2 ± 0.6 s^{-1})
I107 (R_{ex}=7.8 ± 4.0 s^{-1})
H119 (R_{ex}=2.5 ± 1.3 s^{-1})
L121 (R_{ex}=7.1 ± 2.4 s^{-1})
F125 (R_{ex}=11.1 ± 3.3 s^{-1})
Q127 (R_{ex}=4.1 ± 1.6 s^{-1})
I131 (R_{ex}=4.0 ± 2.2 s^{-1})
E135 (R_{ex}=17.6 ± 4.9 s^{-1})
I136 (R_{ex}=11.2 ± 2.6 s^{-1})
G141 (R_{ex}=11.9 ± 2.9 s^{-1})
G143 (R_{ex}=9.8 ± 2.2 s^{-1})
K144 (R_{ex}=6.5 ± 1.6 s^{-1})
G147 (R_{ex}=1.3 ± 0.4 s^{-1})
T152 (R_{ex}=19.0 ± 4.8 s^{-1})
D155 (R_{ex}=4.6 ± 1.3 s^{-1})
H168 (R_{ex}=10.2 ± 2.7 s^{-1})
T169 (R_{ex}=18.4 ± 3.3 s^{-1})
V170 (R_{ex}=1.6 ± 0.8 s^{-1})
N171 (R_{ex}=26.1 ± 14.8 s^{-1})
$R_{2,eff}$ (s^{-1})
ν_{CMPG} (Hz)

C

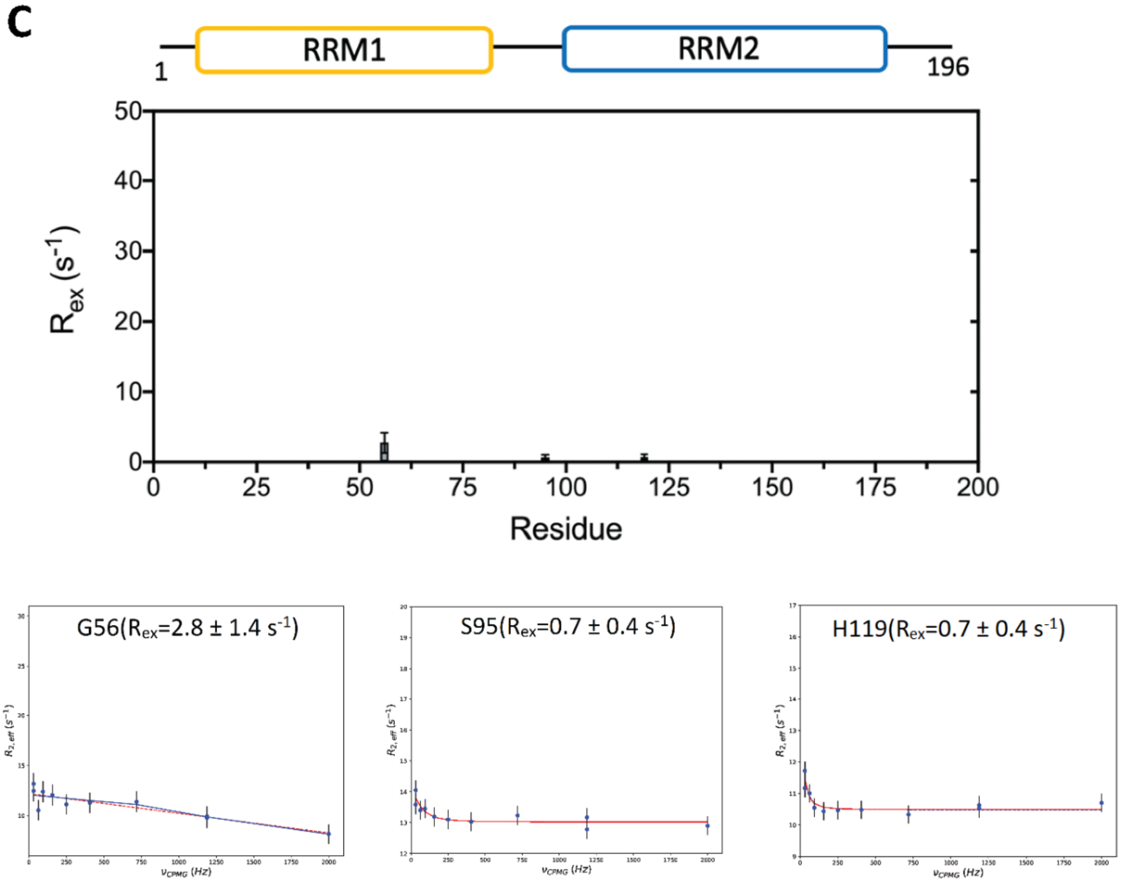

Figure S3. Relaxation dispersion profiles of UP1 and its RRM domains. (**A, B**) Complete ^{15}N relaxation dispersion profiles measured for residues within (**A**) RRM1 and (B**)** RRM2 (**C**) Chemical exchange contribution to the ^{15}N transverse relaxation rate (R_{ex}) measured for amide resonances of the isolated UP1 domain. There was low signal to noise in many of the experiments, resulting in high values for the error bars in RRM2 and instances of divergent replicate measurements.

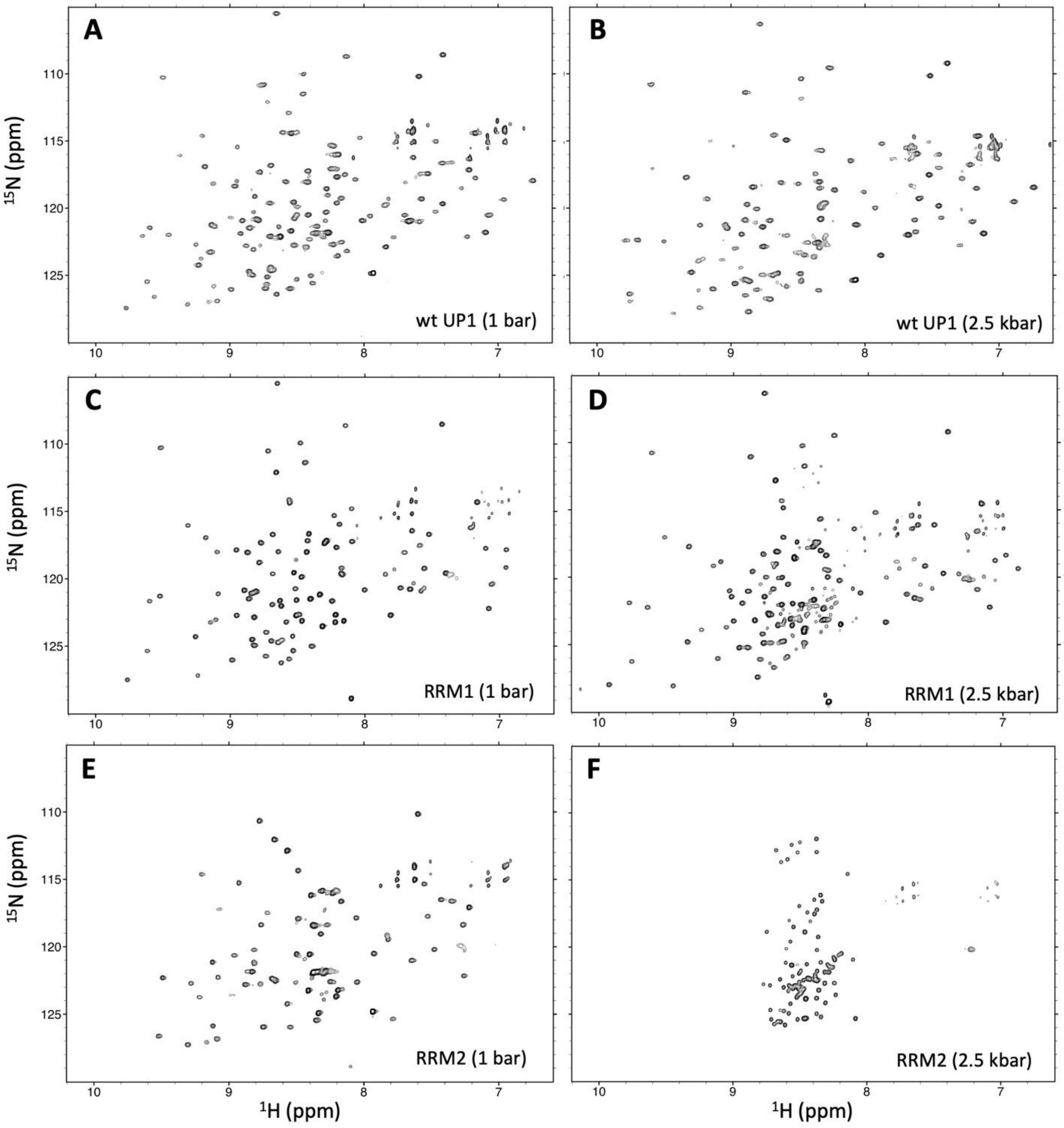
A
B
C
D
E
F
wt UP1 (1 bar)
wt UP1 (2.5 kbar)
RRM1 (1 bar)
RRM1 (2.5 kbar)
RRM2 (1 bar)
RRM2 (2.5 kbar)
15N (ppm)
1H (ppm)
110
115
120
125
10
9
8
7

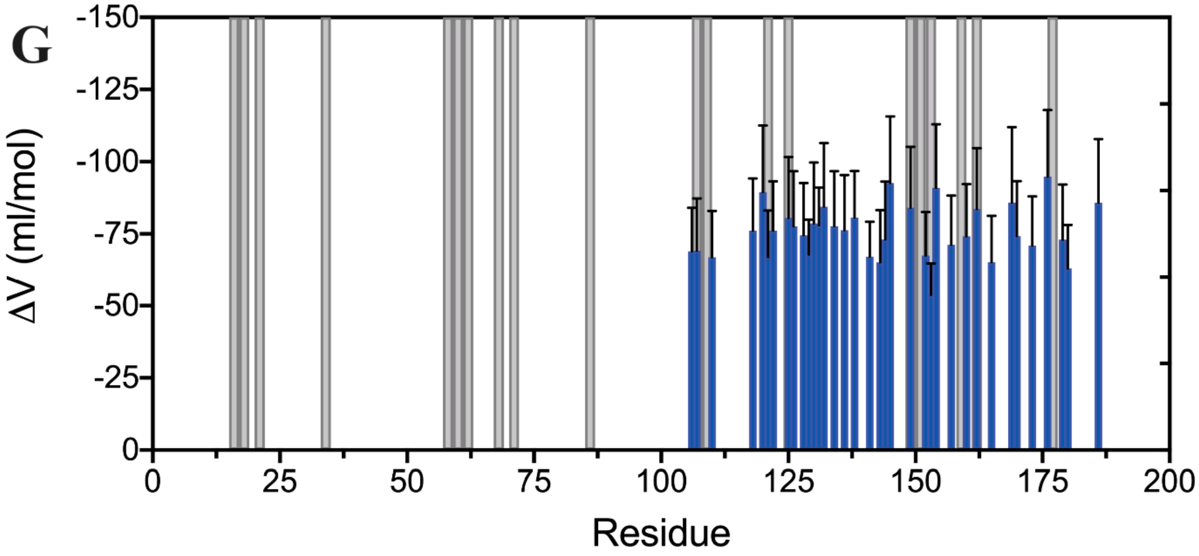

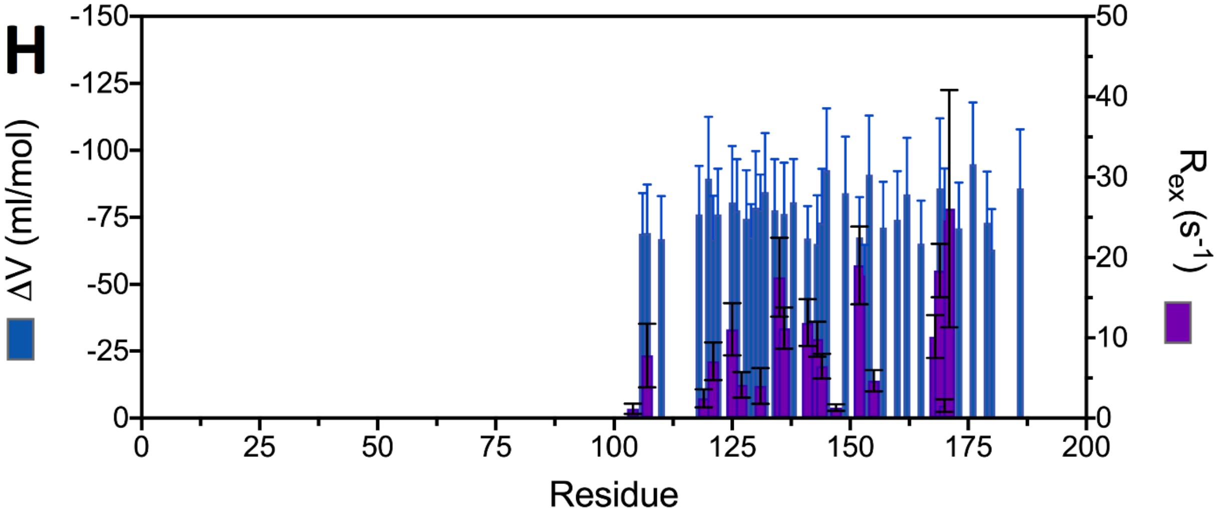

Figure S4. The effect of pressure on UP1 and its RRM domains. ^{15}N-^{1}H HSQC spectra collected at 1 bar and 2.5 kbar for (**A-B**) wt UP1, (**C-D**) the isolated RRM1 motif, and (**E-F**) the isolated RRM2 motif. Spectrum with narrow ^{1}H chemical shift dispersion observed for RRM2 at 2.5 kbar is characteristic of fully disordered protein chains and therefore indicates that RRM2 experiences complete unfolding within this pressure range. (**G**) Individual intensity profiles of RRM2 core residues vs. pressure using a two-state model to calculate dG and dV values. (**H**) Individual intensity profiles of R_{ex} RRM2 residues vs. pressure using a two-state model to calculate dG and dV values.

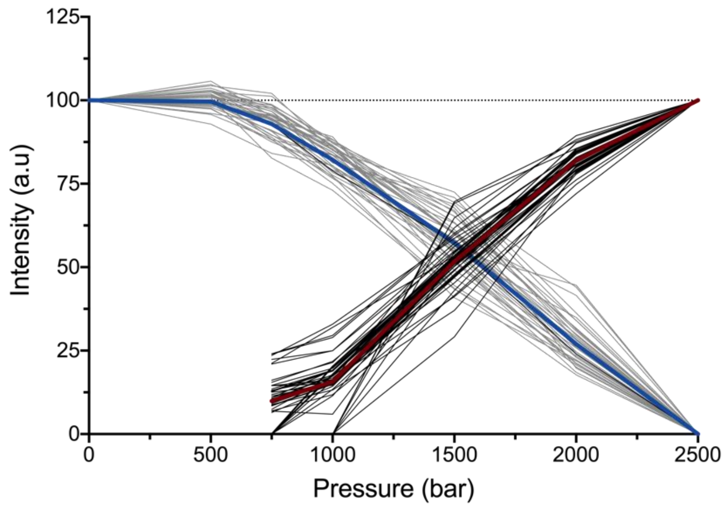

Figure S5. **Conformational transisiton of RRM2.** Comparison of peak intensity profiles measured for amide resonances assigned to residues of RRM2 in a native (folded) state (pale gray lines, average profile shown in blue) with intensity profiles measured residues of RRM2 in a non-native (unfolded) state (black lines, average profile shown in red).

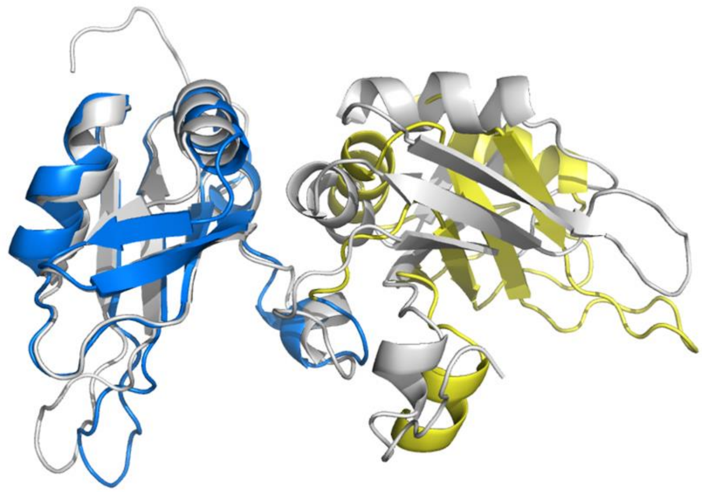

Figure S6. **Inter-RRM orientation of UP1^{swap}.** Comparison of the structure prediction generated by Alphafold2 for the UP1^{swap} variant (with RRM2 in blue and RRM1 in yellow) with the reference high-resolution X-ray structure of UP1 (gray, PDB 1U1R). In the rendering, the structures have been aligned to the RRM1 domains to facilitate the viewing of the relative orientations of RRM2.

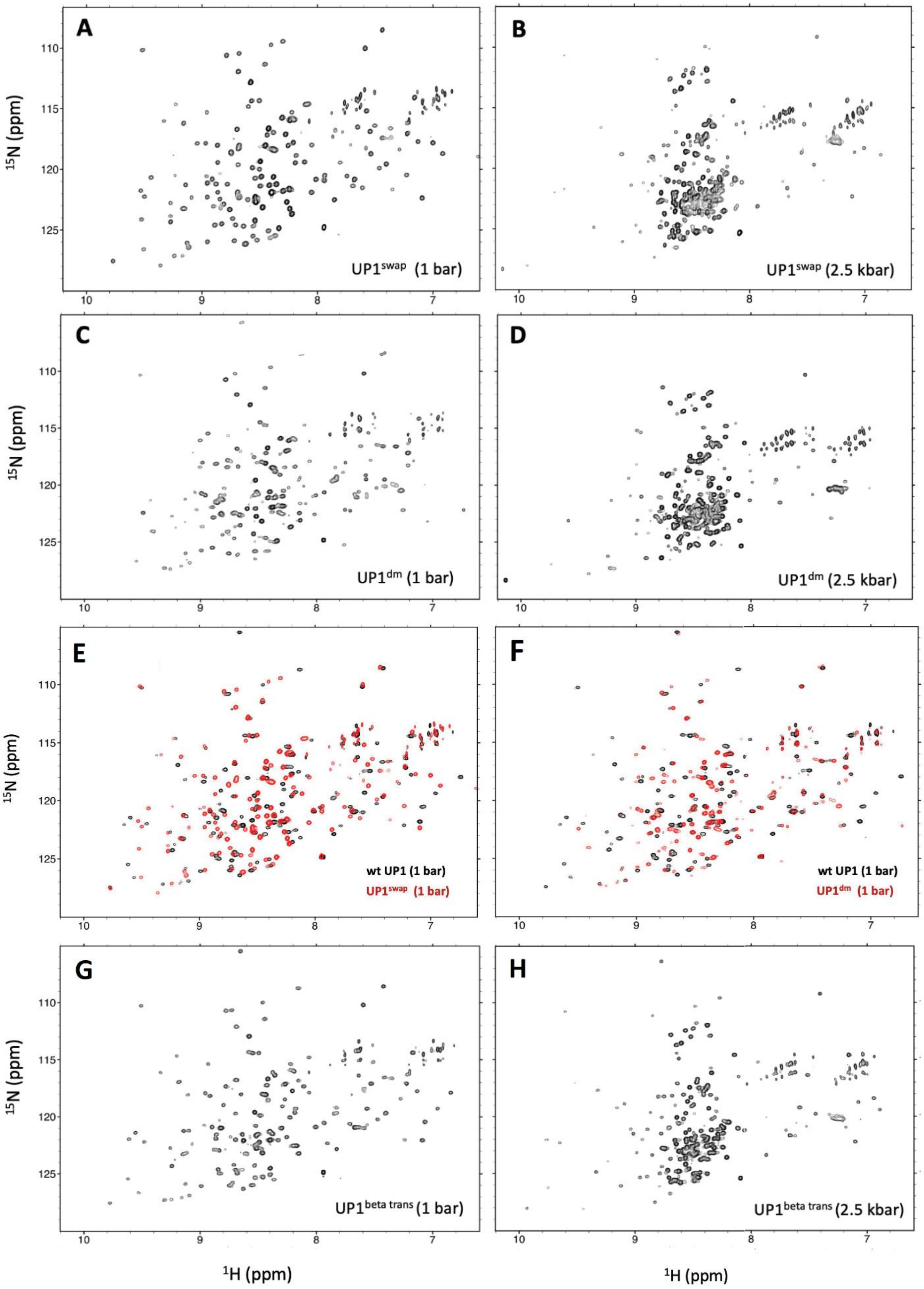

A
B
C
D
E
F
G
H
UP1swap (1 bar)
UP1swap (2.5 kbar)
UP1dm (1 bar)
UP1dm (2.5 kbar)
wt UP1 (1 bar)
UP1swap (1 bar)
wt UP1 (1 bar)
UP1dm (1 bar)
UP1beta trans (1 bar)
UP1beta trans (2.5 kbar)
15N (ppm)
1H (ppm)

Figure S7. Pressure effects for UP1 variants. ^{15}N-^{1}H HSQC spectra collected at 1 bar and 2.5 kbar for (**A-B**) UP1^{swap}, (**C-D**) UP1^{dm}, (**E**) overlay of wt UP1 with UP1^{swap}, (**F**) overlay of wt UP1 with UP1^{dm}, and (**G-H**) UP1$^{beta\ trans}$. All three variants show evidence of significant unfolding under pressure.

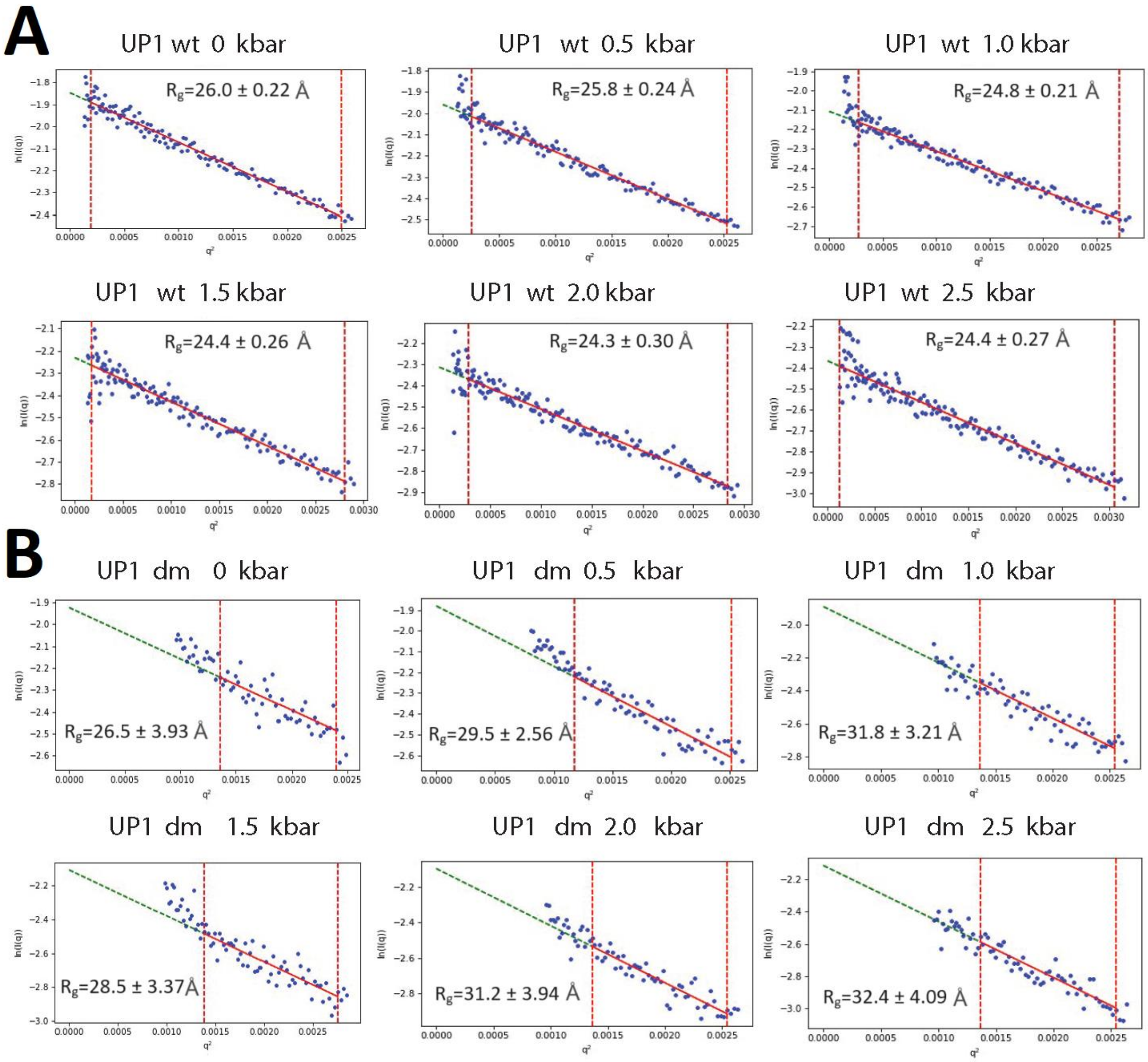

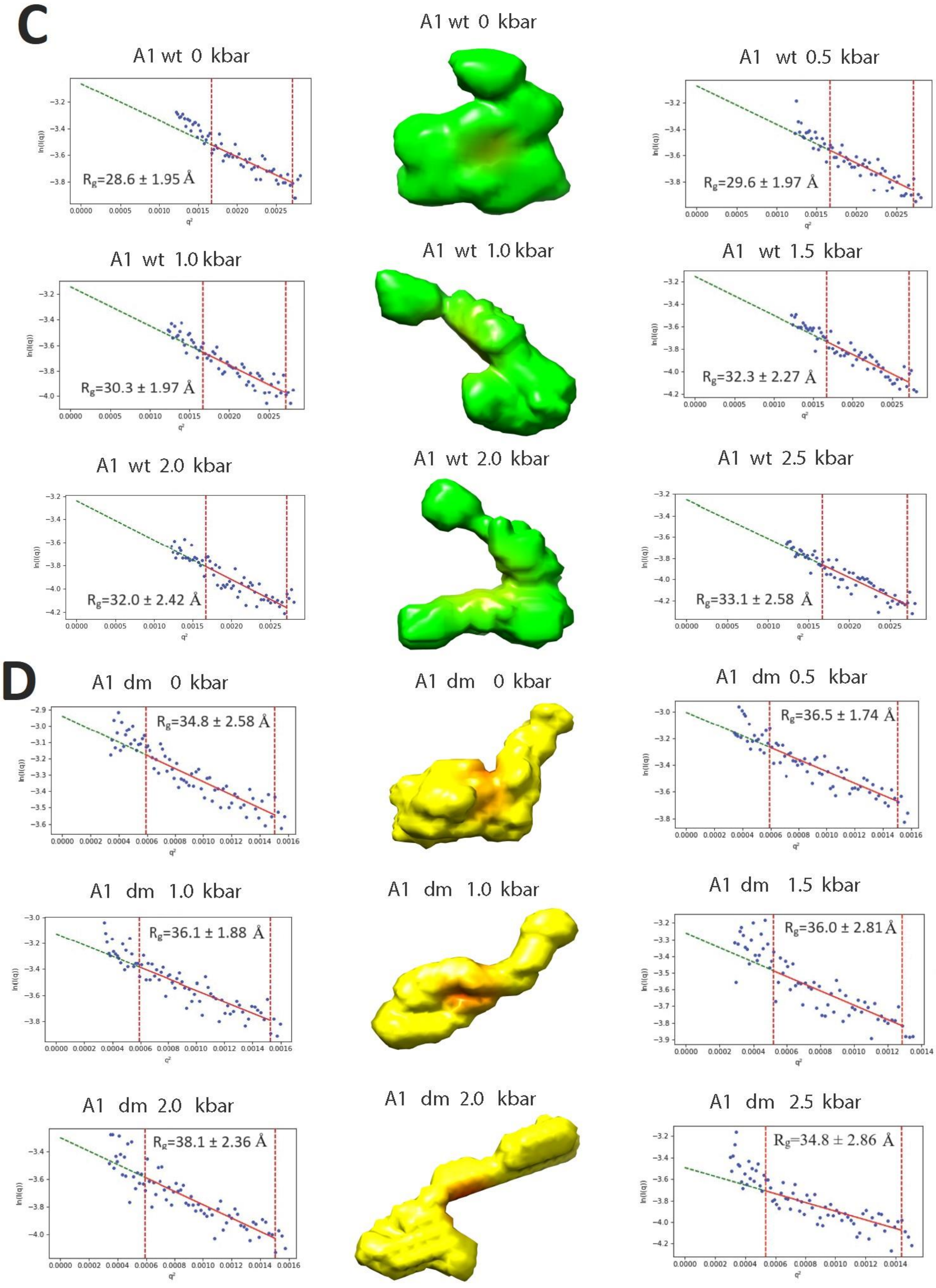
C
A1 wt 0 kbar
A1 wt 0 kbar
A1 wt 0.5 kbar
Rg=28.6 ± 1.95 Å
Rg=29.6 ± 1.97 Å
A1 wt 1.0 kbar
A1 wt 1.0 kbar
A1 wt 1.5 kbar
Rg=30.3 ± 1.97 Å
Rg=32.3 ± 2.27 Å
A1 wt 2.0 kbar
A1 wt 2.0 kbar
A1 wt 2.5 kbar
Rg=32.0 ± 2.42 Å
Rg=33.1 ± 2.58 Å
D
A1 dm 0 kbar
A1 dm 0 kbar
A1 dm 0.5 kbar
Rg=34.8 ± 2.58 Å
Rg=36.5 ± 1.74 Å
A1 dm 1.0 kbar
A1 dm 1.0 kbar
A1 dm 1.5 kbar
Rg=36.1 ± 1.88 Å
Rg=36.0 ± 2.81 Å
A1 dm 2.0 kbar
A1 dm 2.0 kbar
A1 dm 2.5 kbar
Rg=38.1 ± 2.36 Å
Rg=34.8 ± 2.86 Å

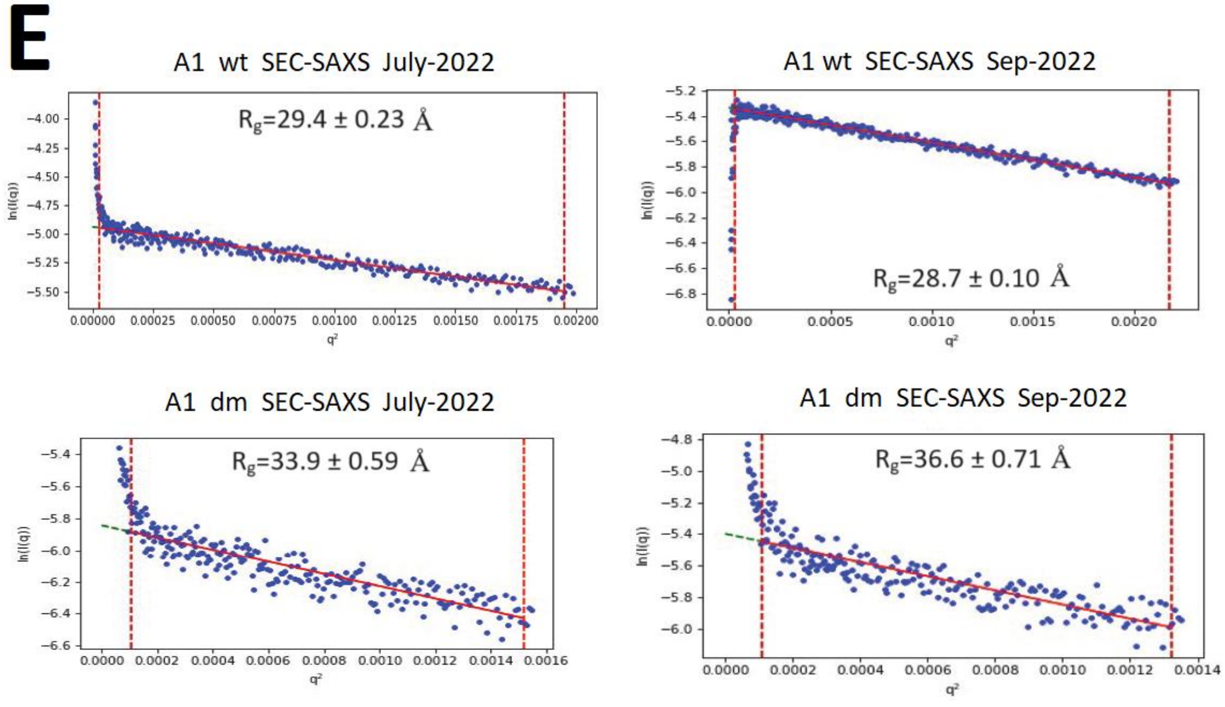

Figure S8. Plots used for R_g calculations from SAXS data. Guinier plots calculated for HP-SAXS experiments and select SAXS envelopes for (**A**) wt UP1, (**B**) UP1^{dm}, (**C**) wt A1, (**D**) Al^{dm}, as well as (**E**) SEC-SAXS. Guinier plots for R_g value calculations were constructed using BioXTAS RAW 2.1.4 *(55)*.

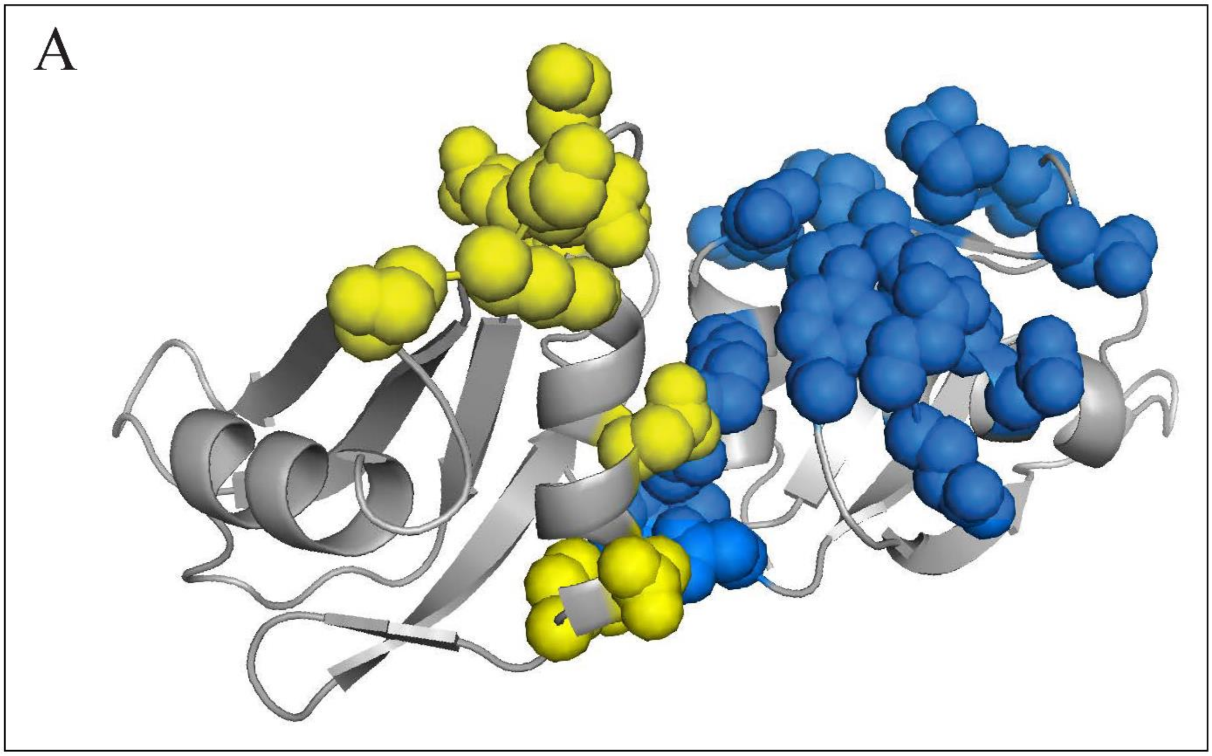
A

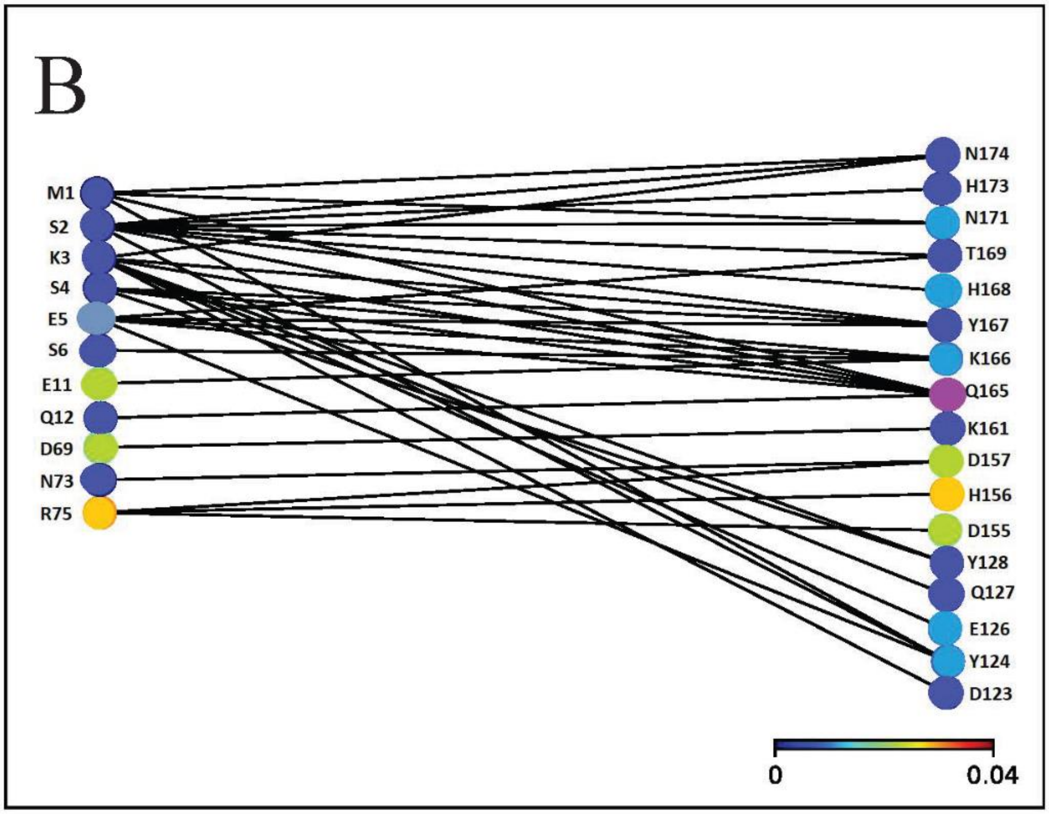
B
M1
S2
K3
S4
E5
S6
E11
Q12
D69
N73
R75
N174
H173
N171
T169
H168
Y167
K166
Q165
K161
D157
H156
D155
Y128
Q127
E126
Y124
D123
0
0.04

C

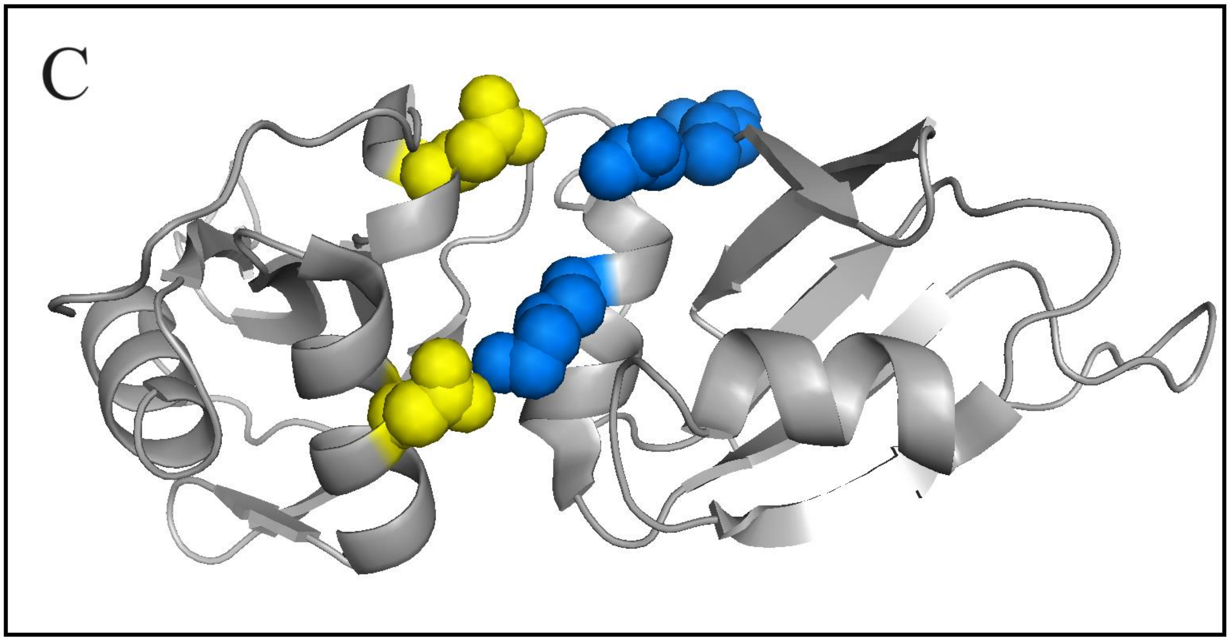

D

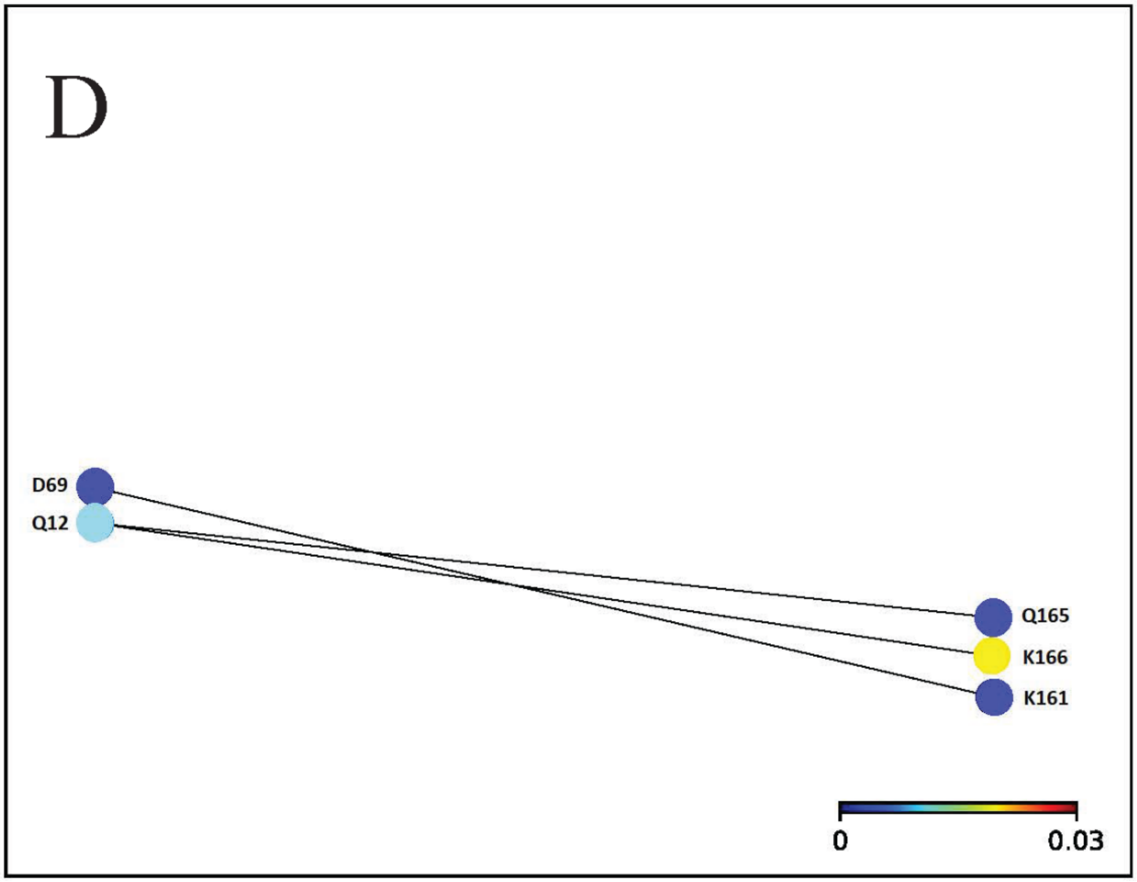

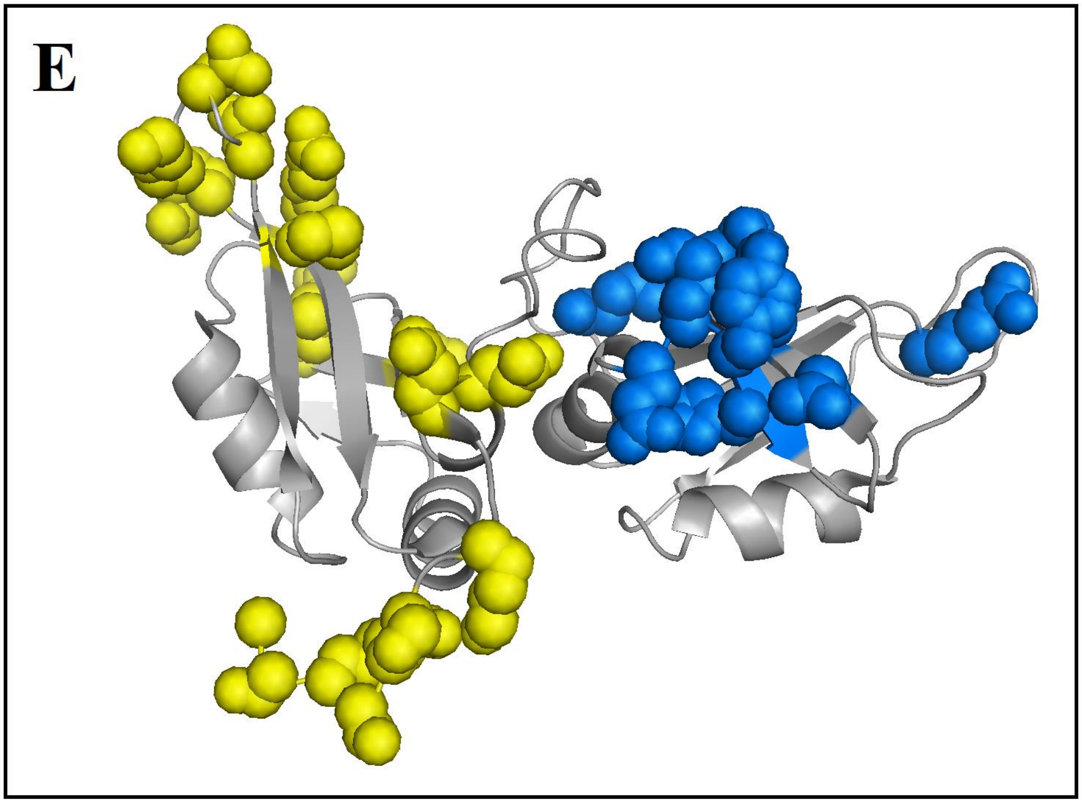
E

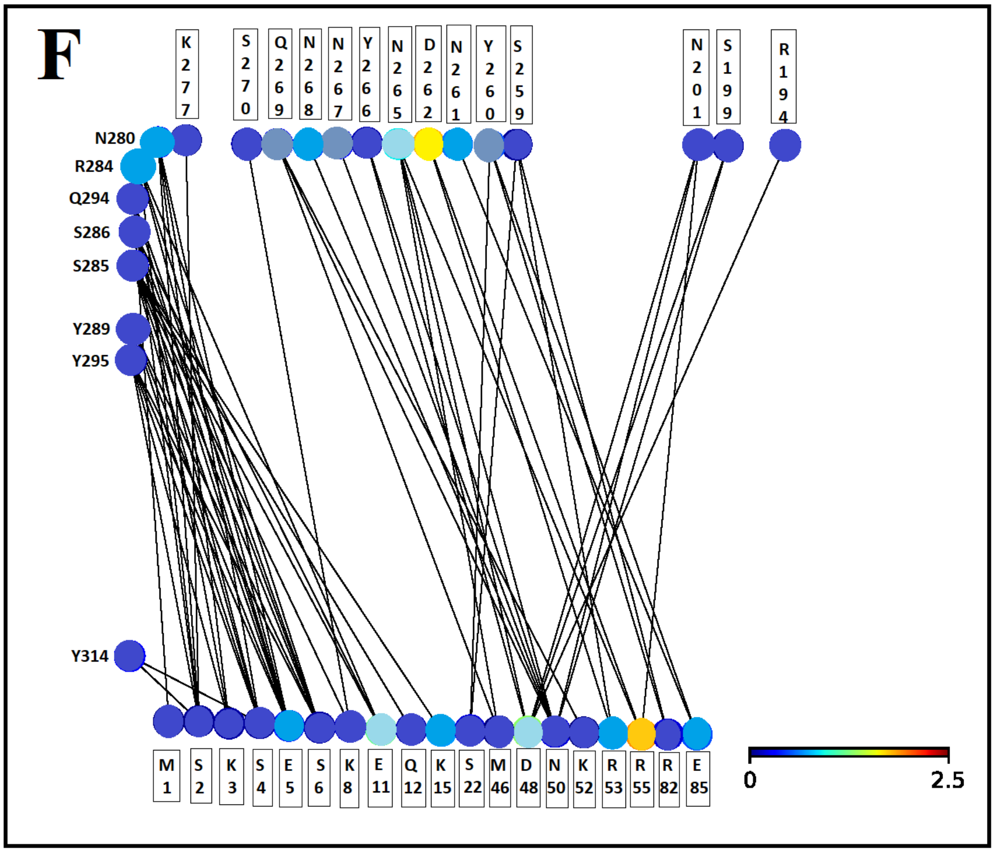
F
K277
S270
Q269
N268
N267
Y266
N265
D262
N261
Y260
S259
N201
S199
R194
N280
R284
Q294
S286
S285
Y289
Y295
Y314
M1
S2
K3
S4
E5
S6
K8
E11
Q12
K15
S22
M46
D48
N50
K52
R53
R55
R82
E85
0
2.5

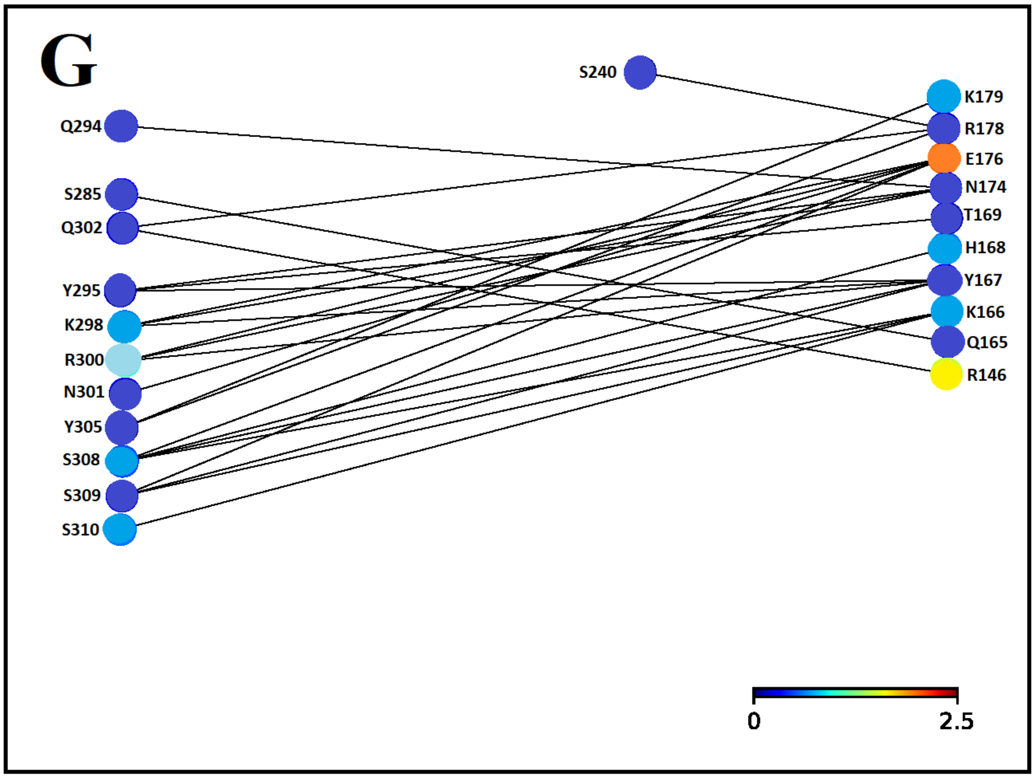
G
S240
Q294
S285
Q302
Y295
K298
R300
N301
Y305
S308
S309
S310
K179
R178
E176
N174
T169
H168
Y167
K166
Q165
R146
0
2.5

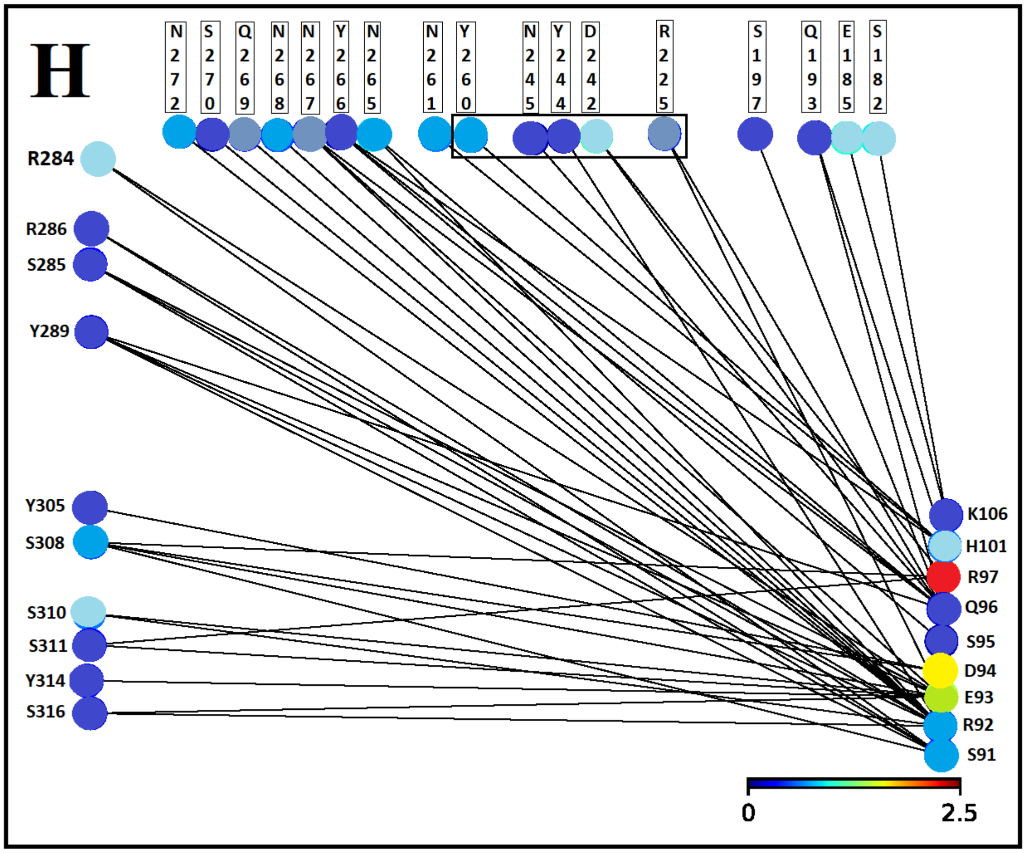
H
N272
S270
Q269
N268
N267
Y266
N265
N261
Y260
N245
Y244
D242
R225
S197
Q193
E185
S182
R284
R286
S285
Y289
Y305
S308
S310
S311
Y314
S316
K106
H101
R97
Q96
S95
D94
E93
R92
S91
0
2.5

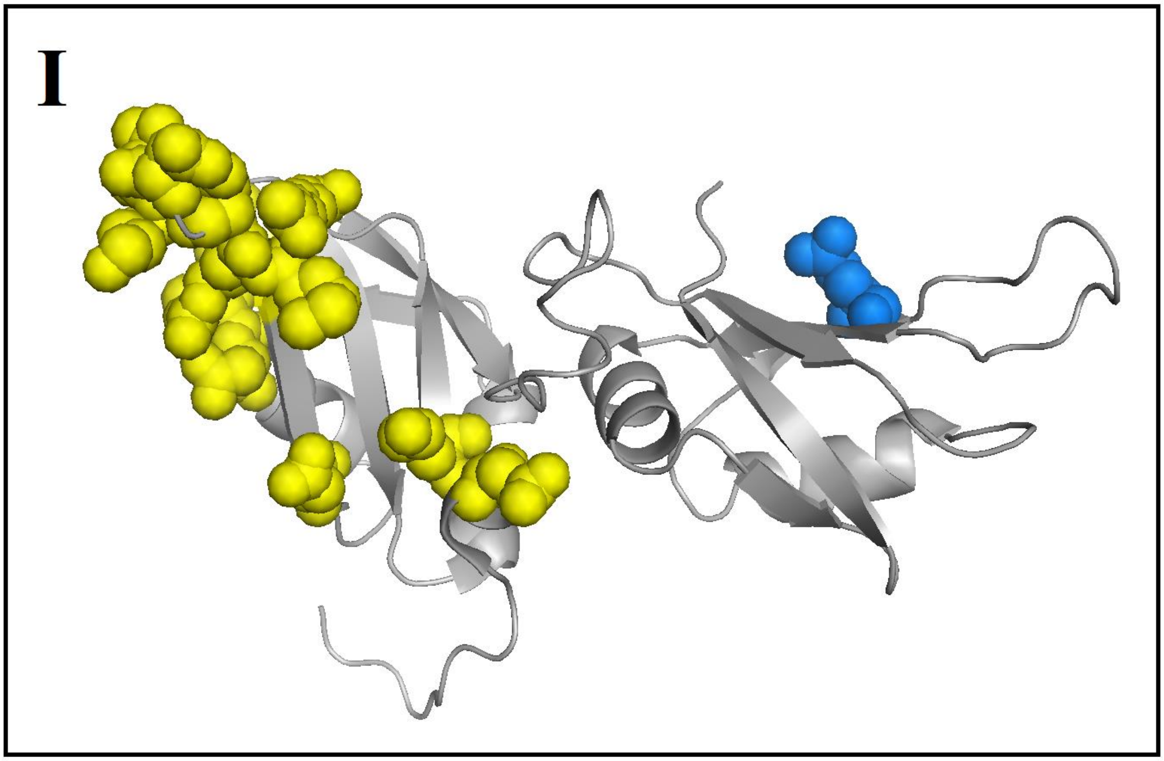
I

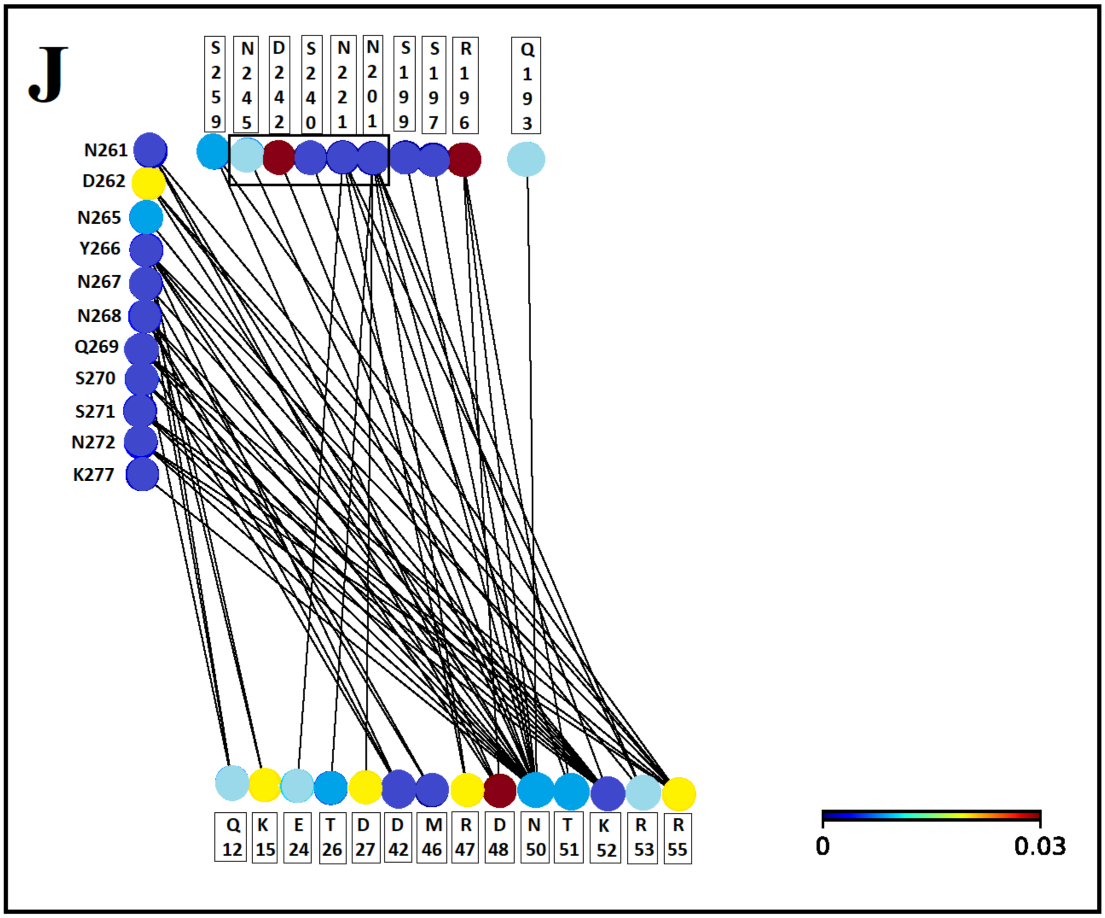
J
S259
N245
D242
S240
N221
N201
S199
S197
R196
Q193
N261
D262
N265
Y266
N267
N268
Q269
S270
S271
N272
K277
Q 12
K 15
E 24
T 26
D 27
D 42
M 46
R 47
D 48
N 50
T 51
K 52
R 53
R 55
0
0.03

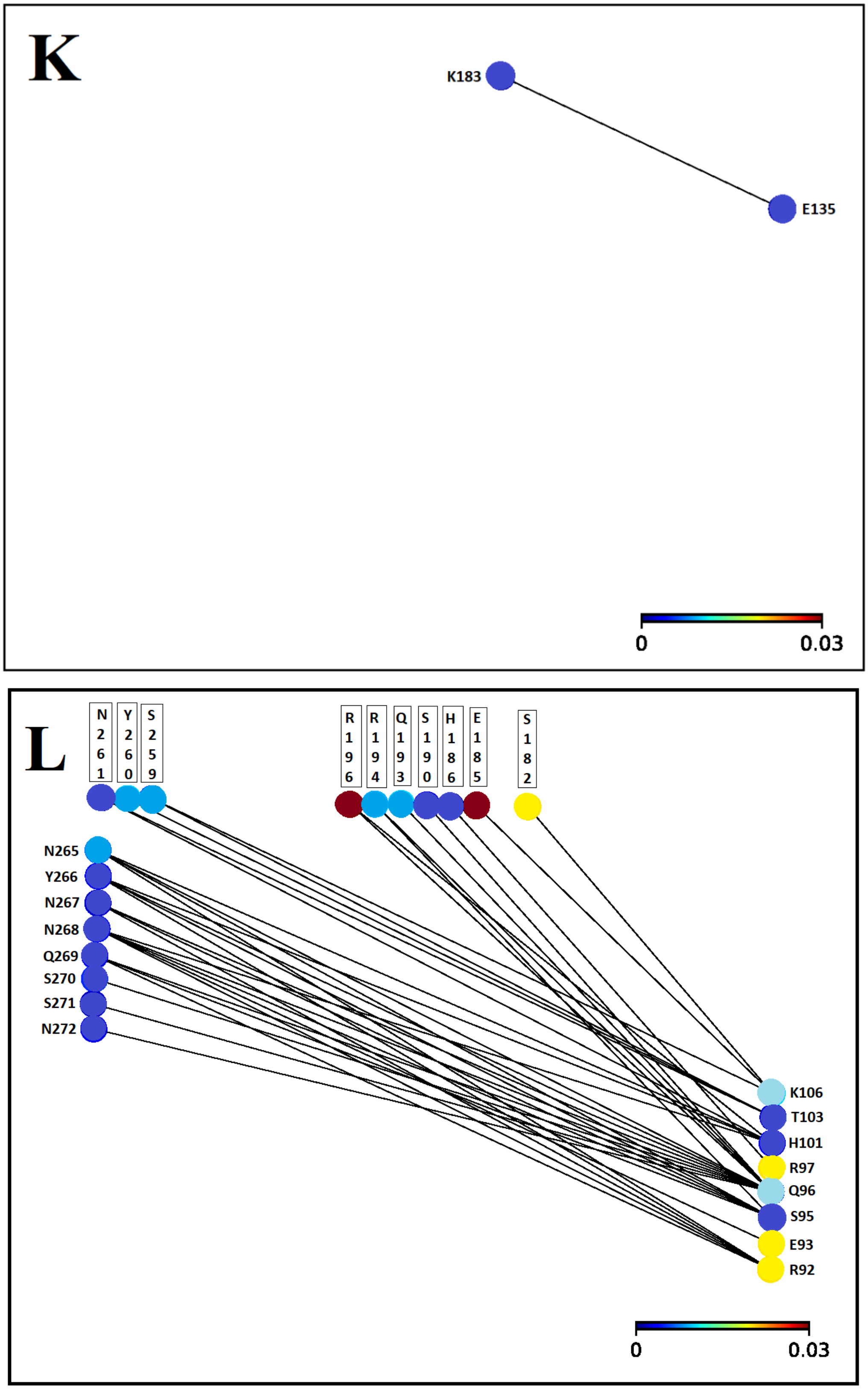

K
K183
E135
0
0.03
L
N261
Y260
S259
R196
R194
Q193
S190
H186
E185
S182
N265
Y266
N267
N268
Q269
S270
S271
N272
K106
T103
H101
R97
Q96
S95
E93
R92
0
0.03

Figure S9: Hbond network as determined by molecular dynamic simulations. (**A**) wt inter-RRM interactions mapped out onto UP1 (**B**) raw data for WT inter-RRM (**C**) UP1^{dm} residues involved in Hbonds mapped onto UP1 (**D**) Raw data for UP1^{dm} inter-RRM interactions (**E**) residues involved in UP1-LCD$_{A1}$ Hbond network for WT RRM1 and RRM2 (**F**) Raw data for WT RRM1-LCD$_{A1}$ interactions (**G**) Raw data for WT RRM2-LCD$_{A1}$ interactions (**H**) raw data for WT inter-RRM linker Hbond interactions with LCD$_{A1}$, RGG box residues are highlighted by the black box (**I**) A1^{dm} residues involved in UP1-LCD$_{A1}$ interactions mapped out on RRM1 (**J**) raw data for A1^{dm} RRM1-LCD$_{A1}$interactions, residues of the RGG box are highlighted by black box (**K**) Raw data for A1^{dm} RRM2-LCD$_{A1}$ interactions, (**L**) raw data for A1^{dm} inter-RRM interactions with LCD$_{A1}$. The amino acids identified are colored by the Betweenness Centrality (BC) values calculated for each residue. BC measures the extent to which each residue interacts with other residues. The scale of these values is indicated by the color bar, and differentiates between WT and dm mutant as the WT proteins have more interactions.

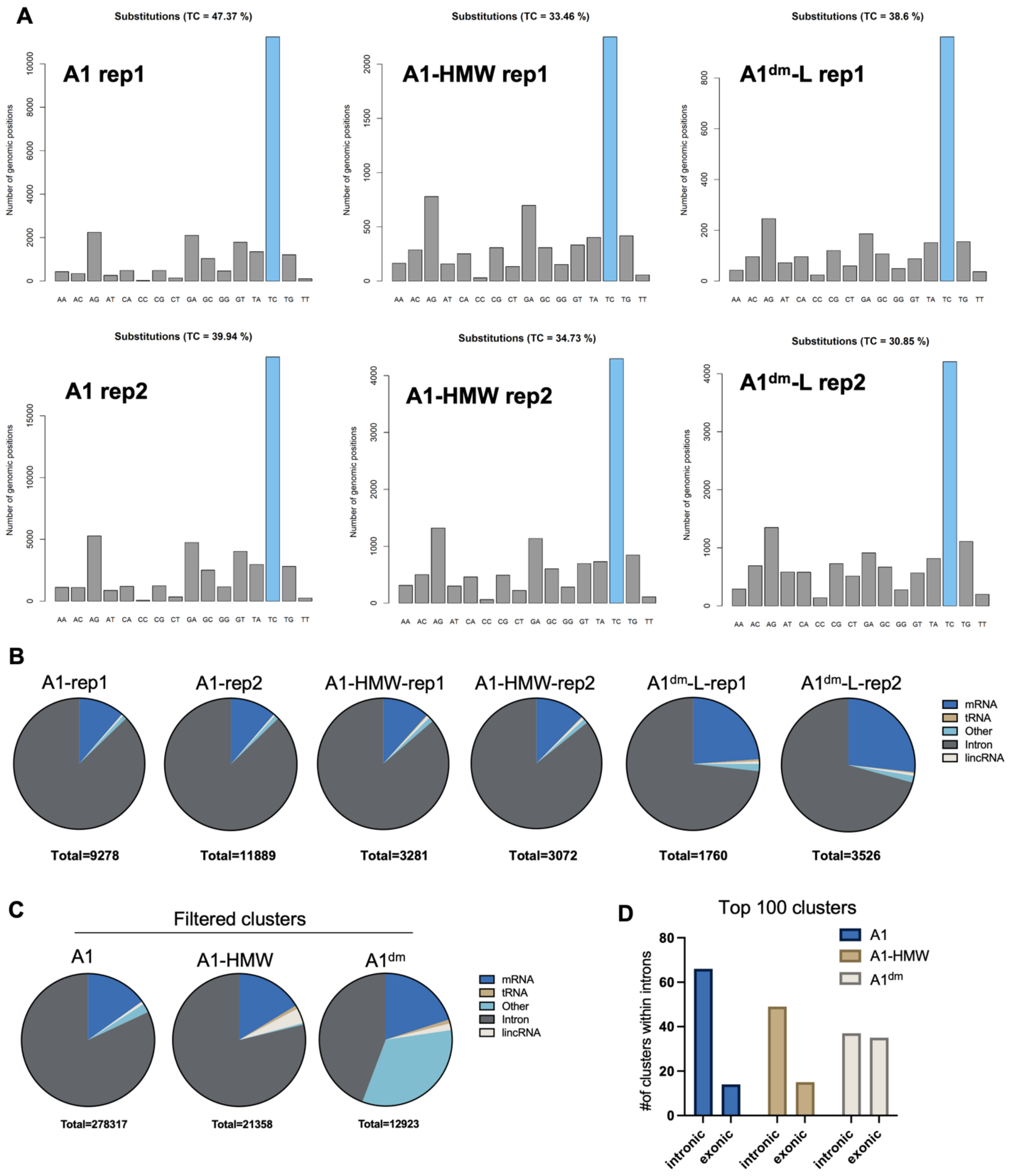

Fig. S10: PAR-CLIP analysis of WT hnRNP A1 and the A1^{dm} mutant. (**A**) Frequency of the indicated nucleotide substitutions in PAR-CLIP-derived reads mapping to the human transcriptome are shown. (**B**) PAR-CLIP reads were formed into “clusters/binding sites” and annotated. Number of clusters that are present in the introns, mRNAs, tRNAs, lincRNAs, miRNAs and other RNA groups are shown. (**C**) PAR-CLIP derived peaks for each hnRNP A1 variant were

filtered to remove those with less than 10 reads and were present only in one replicate. Number of PAR-CLIP reads that map to the indicated RNA species within these filtered peaks are shown. (**D**) Analysis of the number of peaks located in introns vs. exons in the top 100 (sorted by read count) filtered peaks.

Table S2: Accession numbers for high-pressure SAXS and SEC-SAXS experiments deposited in the Small Angle Scattering Biological Data Bank (SASBDB).

High-Pressure SAXS		
Sample	Pressure (Mpa)	Accession Number
UP1 wt	0	SASDT68
	50	SASDT78
	100	SASDT88
	150	SASDT98
	200	SASDTA8
	250	SASDTAB8
UP1 dm	0	SASDTL8
	50	SASDTM8
	100	SASDTN8
	150	SASDTP8
	200	SASDTQ8
	250	SASDTR8
hnRNP A1 wt	0	SASDTY7
	50	SASDTZ7
	100	SASDTs8
	150	SASDT38
	200	SASDT48
	250	SASDT58
hnRNP A1 dm	0	SASDTD8
	50	SASDTE8
	100	SASDTF8
	150	SASDTG8
	200	SASDTH8
	250	SASDTJ8
SEC-SAXS		
Sample	Date	Accession Number
wt hnRNP A1	Jul-22	SASDTW7
	1-Sep	SASDTX7
hnRNP A1 dm	1-Jul	SASDTC8
	22-Sep	SASDTD8

Table S1. (separate file)

CLIP-seq-derived clusters for WT hnRNP A1, hnRNP A1-HMW and hnRNP A1dm
CLIP-seq-derived reads were mapped to the human genome (hg19) and mapped reads were formed into clusters (i.e. binding sites) using PARalyzer. Clusters were subsequently annotated using in-house scripts. Table shows the location and sequence of the clusters, number of reads and T-to-C substitutions present within each cluster and annotation features.
